# Evaluation of Nanopore sequencing for *Mycobacterium tuberculosis* drug susceptibility testing and outbreak investigation: a genomic analysis

**DOI:** 10.1016/S2666-5247(22)00301-9

**Published:** 2023-02

**Authors:** Michael B Hall, Marie Sylvianne Rabodoarivelo, Anastasia Koch, Anzaan Dippenaar, Sophie George, Melanie Grobbelaar, Robin Warren, Timothy M Walker, Helen Cox, Sebastien Gagneux, Derrick Crook, Tim Peto, Niaina Rakotosamimanana, Simon Grandjean Lapierre, Zamin Iqbal

**Affiliations:** a European Molecular Biology Laboratory, European Bioinformatics Institute, Wellcome Genome Campus, Hinxton, UK; b Mycobacteriology Unit, Institut Pasteur de Madagascar, Antananarivo, Madagascar; c Departamento de Microbiología, Medicina Preventiva y Salud Pública, Universidad de Zaragoza, Zaragoza, Spain; d SAMRC/NHLS/UCT Molecular Mycobacteriology Research Unit and DST-NRF Centre of Excellence for Biomedical TB Research, Department of Pathology, University of Cape Town, Cape Town, South Africa; e Division of Medical Microbiology, Department of Pathology, University of Cape Town, Cape Town, South Africa; f Wellcome Centre for Infectious Disease Research in Africa, University of Cape Town, Cape Town, South Africa; g Institute of Infectious Disease and Molecular Medicine, Faculty of Health Sciences, University of Cape Town, Cape Town, South Africa; h Department of Science and Innovation-National Research Foundation Centre for Excellence for Biomedical Tuberculosis Research, SAMRC Centre for Tuberculosis Research, Division of Molecular Biology and Human Genetics, Faculty of Medicine and Health Sciences, Stellenbosch University, Tygerberg, South Africa; i Tuberculosis Omics Research Consortium, Family Medicine and Population Health, Institute of Global Health, Faculty of Medicine and Health Sciences, University of Antwerp, Antwerp, Belgium; j Nuffield Department of Clinical Medicine, John Radcliffe Hospital, Oxford University, Oxford, UK; k Oxford University Clinical Research Unit, Ho Chi Minh City, Viet Nam; l Department of Medical Parasitology and Infection Biology, Swiss Tropical and Public Health Institute, Basel, Switzerland; m University of Basel, Basel, Switzerland; n Department of Microbiology, Infectious Diseases and Immunology, Université de Montréal, Montréal, QC, Canada; o Immunopathology Axis, Centre de Recherche du Centre Hospitalier de l'Université de Montréal, Montréal, QC, Canada

## Abstract

**Background:**

*Mycobacterium tuberculosis* whole-genome sequencing (WGS) has been widely used for genotypic drug susceptibility testing (DST) and outbreak investigation. For both applications, Illumina technology is used by most public health laboratories; however, Nanopore technology developed by Oxford Nanopore Technologies has not been thoroughly evaluated. The aim of this study was to determine whether Nanopore sequencing data can provide equivalent information to Illumina for transmission clustering and genotypic DST for *M tuberculosis*.

**Methods:**

In this genomic analysis, we analysed 151 *M tuberculosis* isolates from Madagascar, South Africa, and England, which were collected between 2011 and 2018, using phenotypic DST and matched Illumina and Nanopore data. Illumina sequencing was done with the MiSeq, HiSeq 2500, or NextSeq500 platforms and Nanopore sequencing was done on the MinION or GridION platforms. Using highly reliable PacBio sequencing assemblies and pairwise distance correlation between Nanopore and Illumina data, we optimise Nanopore variant filters for detecting single-nucleotide polymorphisms (SNPs; using BCFtools software). We then used those SNPs to compare transmission clusters identified by Nanopore with the currently used UK Health Security Agency Illumina pipeline (COMPASS). We compared Illumina and Nanopore WGS-based DST predictions using the Mykrobe software and mutation catalogue.

**Findings:**

The Nanopore BCFtools pipeline identified SNPs with a median precision of 99·3% (IQR 99·1–99·6) and recall of 90·2% (88·1–94·2) compared with a precision of 99·6% (99·4–99·7) and recall of 91·9% (87·6–98·6) using the Illumina COMPASS pipeline. Using a threshold of 12 SNPs for putative transmission clusters, Illumina identified 98 isolates as unrelated and 53 as belonging to 19 distinct clusters (size range 2–7). Nanopore reproduced 15 out of 19 clusters perfectly; two clusters were merged into one cluster, one cluster had a single sample missing, and one cluster had an additional sample adjoined. Illumina-based clusters were also closely replicated using a five SNP threshold and clustering accuracy was maintained using mixed Illumina and Nanopore datasets. Genotyping resistance variants with Nanopore was highly concordant with Illumina, having zero discordant SNPs across more than 3000 SNPs and four insertions or deletions (indels), across 60 000 indels.

**Interpretation:**

Illumina and Nanopore technologies can be used independently or together by public health laboratories performing *M tuberculosis* genotypic DST and outbreak investigations. As a result, clinical and public health institutions making decisions on which sequencing technology to adopt for tuberculosis can base the choice on cost (which varies by country), batching, and turnaround time.

**Funding:**

Academy for Medical Sciences, Oxford Wellcome Institutional Strategic Support Fund, and the Swiss South Africa Joint Research Award (Swiss National Science Foundation and South African National Research Foundation).

## Introduction

Ten years of progress in reducing the global burden of tuberculosis have been lost because of the SARS-CoV-2 pandemic, with 1·4 million fewer patients diagnosed and treated in 2020 than in 2019.^1^, ^2^ Accurate diagnosis and appropriate treatment are key to setting the global effort to end tuberculosis back on course.^3^ Understanding and interrupting transmission are equally important, as is implementing appropriate therapy for every patient. In high-income settings, whole-genome sequencing (WGS) has become a solution to both these challenges, with some health systems now relying predominantly on WGS for drug susceptibility testing (DST) and implementation of individualised therapeutic regimens,^4^, ^5^ in addition to the well documented benefits of using these data for surveillance and outbreak control.^6^

With the availability of multiple DNA sequencing platforms, simplified access to interpretation of sequencing data,^6^ and curated genomic databases,^7^ more countries and health initiatives are now integrating DNA sequencing within tuberculosis control programmes for either or both DST and epidemiological surveillance.^5^

**Research in context**

**Evidence before this study**

We searched PubMed on Feb 1, 2022, using the terms “Mycobacterium tuberculosis”, “drug resistance prediction”, “drug susceptibility prediction”, “genome”, “genomic”, and “genotypic” for articles published between Jan 1, 2008, and Feb 1, 2022. No language restrictions were applied to the search. Two key types of information can be obtained from laboratory testing of *Mycobacterium tuberculosis* isolates to help directly guide public health interventions: drug susceptibility testing (DST) to guide therapy, and bacterial typing to enrich understanding of the epidemiology and guide interventions to mitigate transmission. DST is typically performed by the gold standard culture-based phenotyping method or nucleic acid amplification assays targeting specific resistance-conferring mutations. Studies over the past 7 years have shown that prediction of susceptibility profile using Illumina-technology genome sequence data is possible, and can be automated. In a key publication, the CRyPTIC consortium and UK 100,000 Genomes project evaluated the method on over 10 000 genomes including prospectively sampled isolates and showed that for first-line tuberculosis drugs (isoniazid, rifampicin, ethambutol, and pyrazinamide), a pan-susceptibility profile is accurate enough to be used clinically. The genetic basis of resistance remains imperfectly understood for second-line tuberculosis drugs, in particular for new and repurposed drugs (bedaquiline, clofazimine, delamanid, and linezolid). Previous work in the field of genotypic DST was heavily based on Illumina technology, which provides short (70–300 bp) sequence reads of very high quality. Many different softwares (eg, TBProfiler, Mykrobe, MTBseq, and kvarq) have been designed for sequence analysis and genotypic DST. However, the increasingly used Nanopore sequencing platforms yield very different data with much longer sequence reads (frequently over 1 kb) and higher error rates including systematic biases. Only two of these tools can operate with Nanopore data: Mykrobe and TBProfiler. However, the Nanopore-specific parameters for these tools were calibrated on small datasets (n=5 spiked samples of *Mycobacterium bovis* for Mykrobe and 34 replicates from three independent isolates TBProfiler). To date, very limited evaluation of Nanopore-based drug susceptibility prediction has been performed using these two tools (n=22 isolates by Peker and colleagues). Smith and colleagues recently assessed Nanopore-based drug resistance prediction (and clustering) from 431 isolates, but used custom, in-house catalogues and scripts, making it harder for others to reproduce. Molecular typing of *M tuberculosis* allows lineage identification and detection of putative transmission clusters. In the last decade, multiple *M tuberculosis* molecular epidemiology studies have shown how genomic information can complement traditional epidemiology in identifying person-to-person transmission clusters with a high level of resolution. Typically, the number of single nucleotide polymorphism (SNP) disagreements between genomes, or SNP distance, is calculated and single-linkage clustering is performed for genomes falling within retrospectively established transmission thresholds of either five or 12 SNPs. Just as with DST, these thresholds were established with Illumina sequencing data. The increased error rate in Nanopore sequencing is believed to lead to inflated SNP distances if standard genome analysis tools are used. Before this study it was unknown what impact on isolate-clustering this would incur.

**Added value of this study**

Full-scale adoption of genomic sequencing in tuberculosis reference laboratories has so far taken place in a small number of settings (England, the Netherlands, and New York State), all using Illumina-based sequencing data. Building on current evidence, specific WHO technical guidance, and diversification and democratisation of technology, sequencing is expected to be increasingly used in tuberculosis control globally. For the first time, our study offers four key deliverables intended to inform adoption of Nanopore technology as an alternative, or a complement, to Illumina. First, a systematic head-to-head comparison of Nanopore and Illumina data for *M tuberculosis* drug susceptibility profiling and isolate clustering, including quantitative metrics for cluster precision and recall. Second, an assessment of the impact of mixed Illumina and Nanopore data on clustering, which represents an increasingly common challenge faced by public health laboratories in the context of multi-laboratory and sometimes multi-country investigations. Third, an open-source software pipeline allowing research and reference laboratories to replicate our analytical approach. Fourth, a publicly available curated test set of 151 isolates, including matched Illumina and Nanopore sequence data, and (for a subset of seven isolates) high-quality PacBio assemblies, for method development and validation.

**Implications of all the available evidence**

Catalogues of drug resistance-conferring mutations will keep improving, especially for new and repurposed drugs. Our data confirm that Illumina and Nanopore sequencing technologies can be used to identify those mutations equally accurately in *M tuberculosis*. Bacterial molecular typing is constantly shown to support the understanding of disease transmission and tuberculosis control in new settings. The bioinformatics tools and filters we have developed, assessed, and made publicly available allow the use of Nanopore or mixed-technology data to appropriately cluster genetically related isolates. We provide a measure of the expected level of over-clustering associated with Nanopore technology. For reference laboratories performing *M tuberculosis* genotypic DST and cluster-identification, this study supports the adoption of Nanopore sequencing technology and confirms its compatibility with already established Illumina platforms moving forward.

Illumina sequencing platforms are frequently used in public health laboratories and are the established reference standard for tuberculosis WGS. Per-base sequencing accuracy is extremely high, making this technology an attractive tool for both genotypic resistance prediction and for surveillance, whereby just a few erroneous basecalls can be the difference between triggering public health interventions or not. Substantial validation and accreditation work has led to integration of this technology within routine clinical diagnostics in some settings (eg, the UK, the Netherlands, and New York State in the USA). Illumina technology requires large capital outlay and a large testing volume to ensure clinically appropriate turn-around times while remaining cost-efficient. By comparison, Oxford Nanopore Technologies (ONT) offer a more transportable Nanopore-based solution in the form of their handheld MinION sequencing platform, which allows rapid sequencing of the genomes of individual isolates without the delay associated with batching samples from multiple patients. To date, a major obstacle for ONT's technology has been its basecalling error rate. However, as the technology has matured and its basecalling software has improved, it is now increasingly integrated in public health laboratories.^8^, ^9^

To date, a few studies have evaluated the accuracy of nanopore-based genotypic DST.^9^, ^10^, ^11^, ^12^ However, the impact of this sequencing technology on the clustering of isolates in the context of tuberculosis outbreak investigation remains poorly understood.^13^ In this study, we directly compare Nanopore's performance to Illumina platforms and assess whether the accuracy of its outputs has improved sufficiently to justify its use for patient care and public health.

## Methods

### *Mycobacterium tuberculosis* clinical isolates

208 *M tuberculosis* isolates, which were collected between 2001 and 2018, were selected from three countries: Madagascar (2012–17; n=109), South Africa (2011–17; n=67), and England (2017–18; n=32).

The 109 Madagascan isolates were collected as part of the TB-MR national drug resistance surveillance programme and confirmed as multidrug resistant tuberculosis by culture, and were retrospectively included together with a 1:1 matched drug susceptible sample from the same sampling dates and geographical region. For ten patients, isolates from a second positive sample corresponding to the 2-month treatment were also included.

The 67 South African biobanked isolates were from patients routinely diagnosed with rifampicin-resistant tuberculosis in the Western Cape Province.

The 32 English isolates were from the England National Mycobacteria Reference Service in Birmingham, and were selected from routine sequencing of mycobacterial isolates. In all locations this study involved only accessing stored bacterial cultured isolates (appendix pp 5–6), and not directly obtaining or processing human samples.

This study used anonymised pre-existing retrospective collections of isolates and our analyses led to no clinical intervention; no human data were used in this study. Institutional review board approval was therefore not required for this study in Madagascar, South Africa, or England. Nevertheless, the South African data are part of a biobank, for which there is ethics approval for storage of specimens and sequencing (N09/11/296), and linking clinical data to sequence data from the University of Cape Town (416/2014), but this linkage was not done in this study, for which all data were anonymised.

### WGS, data preparation, and quality control

WGS on each isolate was done on both Nanopore and Illumina platforms using extracted DNA from the same bacterial culture (appendix pp 5–7). Illumina sequencing was performed as per the manufacturer's instruction on either the MiSeq (English samples), HiSeq 2500 (Malagasy and South African samples), or NextSeq500 (South African samples) platforms. Nanopore sequencing was performed using the Ligation Sequencing Kit 1D (SQK-LSK108 or SQKLSK109) and the Native Barcoding Kit 1D (EXP-NBD103 or EXP-NBD104) according to the manufacturer's instructions on either the MinION (Malagasy, English, and South African samples) or GridION (English samples) platform with R9·4·1 flow cells. Additionally, 35 Malagasy isolates, including drug-resistant strains, were sequenced on the PacBio CCS platform. After decontamination by aligning reads to a database of contaminants (appendix p 8), isolates with mean read depth less than 20 (Illumina) or 30 (Nanopore) were excluded from the study.^13^

### Variant calling

Illumina single nucleotide polymorphism (SNP) calls were made with the COMPASS pipeline^14^ (appendix p 9) used by the UK Health Security Agency (UKHSA).^5^ Nanopore SNP calls were made using BCFtools (v1.13; appendix pp 9–10).^15^ Repetitive regions of the genome were predefined and these were masked for both technologies (appendix p 9).

### Evaluation of variant precision and recall

We evaluated the precision and recall of the SNP calls for isolates with PacBio truth assemblies (n=7; appendix pp 10–11). We defined precision as the proportion of SNP calls that are true positives and recall as the proportion of expected (true) SNP calls correctly identified. Filters were chosen to optimise two constraints. First, following the UKHSA COMPASS approach, to maximise precision without too much loss of recall, based on the evaluation with the PacBio truth assemblies. Second, to maximise the correlation between the pairwise SNP distances as measured by Illumina, and those measured by Nanopore. Details of the correlation analysis and code are in the appendix (p 18).

### Assessing clusters based on SNP thresholds

We used the pairwise distance matrix (appendix p 10) to assess the impact of sequencing technologies on commonly used SNP thresholds for isolate clustering. For a given SNP threshold *t*, we constructed a clustering network (graph) by connecting isolates with a distance of *t* or less. That is, a cluster is a subgraph within which a path exists between any two isolates, but no path exists to any isolates in another cluster. With this definition, all clusters had a minimum of two members. Isolates that did not cluster with any others are deemed singletons.

Because we sought to show concordance of Nanopore data with UKHSA's Illumina-based strategy, we investigated SNP threshold values of five and 12.^16^ Our goal was to establish whether Nanopore data can be used to reproduce equivalent clusters to those generated with Illumina data. We therefore treated Illumina as the established standard (truth) when comparing clustering. We established three metrics for assessing cluster similarity (appendix pp 11–14). Sample-averaged cluster recall (SACR) indicates whether isolates have been missed by Nanopore clustering (false negatives) and sample-averaged cluster precision (SACP) reflects additional isolates being clustered by Nanopore (false positives). SACR and SACP did not account for Nanopore clusters composed solely of Illumina singletons, so we defined the excess clustering rate (XCR) as the proportion of Illumina singletons that were clustered by Nanopore. A value of 0·1 indicated that 10% of Illumina singletons were part of a Nanopore cluster.

### Simulation of isolate clusters with mixtures of sequencing modalities

To model the impact of using distinct sequencing platforms when supporting epidemiological investigations, we simulated mixed technology datasets by randomly choosing a technology for each isolate. We used Nanopore-to-Illumina ratios of 1:99, 1:19, 1:9, 1:3, 1:1, 3:1, and 9:1. For each ratio and SNP threshold combination we performed the following 1000 times: (1) randomly assigned isolates to a technology in the relevant ratio, and (2) calculated SACR, SACP, and XCR for the relevant SNP threshold.

### Phenotypic DST

Phenotypic DST data were generated by Malagasy and South African laboratories according to local routine protocols (appendix pp 14–16).^17^ For the Malagasy isolates, the indirect proportion method on Löwenstein-Jensen medium was performed to test susceptibility to streptomycin (critical concentration 4·0 μg/mL), isoniazid (0·2 μg/mL), rifampicin (40·0 μg/mL), ethambutol (2·0 μg/mL), kanamycin (30·0 μg/mL), amikacin (30·0 μg/mL), and capreomycin (40·0 μg/mL). For the South African isolates, all phenotypic DST was done on Middlebrook 7H with concentrations of 0·2 μg/mL for isoniazid, 2·0 μg/mL for ofloxacin, and 4·0 μg/mL for amikacin. Phenotypic DST is no longer routinely done by UKHSA.

### Drug resistance prediction from sequencing data

We used Mykrobe (version 0.10.0) to obtain predictions of each isolate's drug susceptibility profile for 11 drugs (appendix pp 17–18).^10^ Mykrobe genotypes sequencing reads against a catalogue of resistance-conferring mutations. This process is independent of the variant calling steps outlined for isolate clustering. The catalogue of resistance mutations used by Mykrobe consists of 476 SNPs defined at the amino acid level (which translates into 3352 at the nucleotide level), 60 promoter SNPs, and 1904 nucleotide-level SNPs, insertions, and deletions (dominated by those in the rifampicin resistance determining region of the gene *rpoB*). Additionally, to detect isoniazid and pyrazinamide resistance-causing frameshifts in the genes *katG* and *pncA*, the catalogue contains an explicit list of all possible 1–2 bp frameshifts in those two genes, totalling 61 258.^10^ We chose not to use the mutation catalogue from WHO,^7^ which was published towards the end of this study in March, 2022, as there was no Mykrobe version of it yet, and the purpose of this study is to determine whether Nanopore genotypes of resistance mutations are consistent with Illumina, which is independent of the catalogue.

### Statistical analysis

After applying filters, the SNP distances between all pairs of isolates as measured by COMPASS (Illumina) and BCFtools (Nanopore) are significantly correlated (*R*^2^=0·988, p<0·0001; appendix pp 18–19). Evaluating concordance of Illumina and Nanopore genotypes and drug resistance variants, genotypes were identical at more than 3000 SNPs each genotyped on 151 samples (100% concordant, Cohen's κ statistic=1, 95% CI [1,1]). There were four discrepancies in total across more than 60 000 indels each genotyped in 151 samples (>99·99% concordant, Cohen's κ statistic=1, 95% CI [1,1]). Further details are in the appendix (p 17). Evaluating concordance of Illumina and Nanopore drug resistance predictions found a Cohen's κ statistic of 0·9915 (p<0·0001).

### Role of the funding source

The funders of the study had no role in study design, data collection, data analysis, data interpretation, or writing of the report.

## Results

Of the 208 *M tuberculosis* isolates, 57 (27%) isolates did not pass quality control measures. Of these 57 isolates, 44 (77%) had insufficient depth: 37 (84%) of 44 on Nanopore, one (2%) of 44 on Illumina, and six (14%) of 44 on both technologies. For 12 (21%) of the 57 isolates, a single lineage call could not be determined. Additionally, one isolate was found to have non-matched Illumina and Nanopore data, probably due to a labelling mix-up. Therefore, 151 isolates sequenced on both Illumina and Nanopore platforms passed quality control: 91 from Madagascar, 41 from South Africa, and 19 from England. Seven from Madagascar had associated PacBio data (appendix p 9).

**Figure 1:**
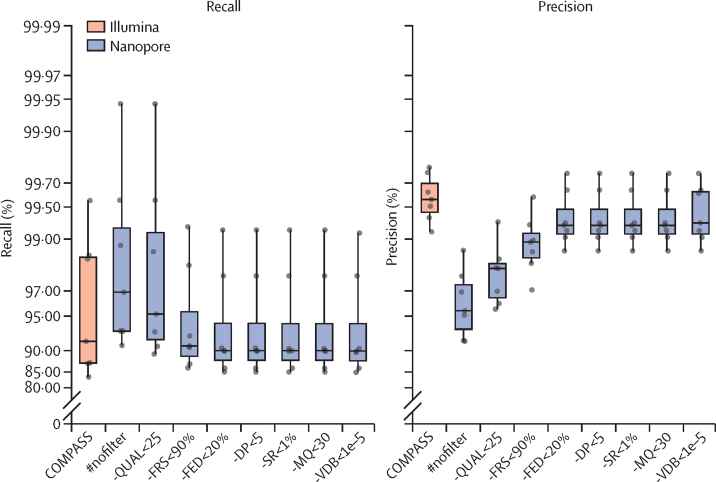
Recall and precision of SNPs from the Illumina COMPASS pipeline, and the Nanopore BCFtools pipeline with a cumulative selection of filters Each point represents a single isolate with a PacBio assembly. The midline in each box plot is the median, the upper and lower bounds of each box indicates the span of the quartiles of the data (ie, IQR), and the whiskers extend 1·5 times the IQR. #nofilter is BCFtools with no filtering of variants. Moving right from #nofilter, each box accumulates a new filter plus the previous ones. Each filter describes the criterion for removing an SNP. -QUAL<25 removes SNPs with a quality score less than 25; -FRS<90% removes SNPs whereby less than 90% of reads support the called allele; -FED<20% removes SNPs with read depth below 20% of the isolate's median depth; -DP<5 removes SNPs with less than five reads at the position; -SR<1% removes SNPs with less than 1% of read depth on either strand; -MQ<30 removes SNPs with a mapping quality below 30; -VDB<1e–5 removes SNPs with a variant distance bias less than 0·00001. SNP=single-nucleotide polymorphism.

The seven isolates with PacBio truth assemblies (appendix p 11) allowed us to assess variant-calling filter thresholds and achieve different balances of precision versus recall. Because our goal was to determine whether Nanopore could be used as an alternative (or complement) to existing Illumina-based pipelines, including COMPASS used by UKHSA, we sought to match their approach, prioritising precision over recall, with the effect of applying successive filters (appendix pp 9–10), shown in figure 1. The final set of filters resulted in a median SNP precision of 99·3% (IQR 99·1–99·6) and recall of 90·2% (88·1–94·2) for the seven validation isolates. By comparison, Illumina data processed with COMPASS had a median precision of 99·6% (99·4–99·7) and recall of 91·9% (87·6–98·6).

**Figure 2:**
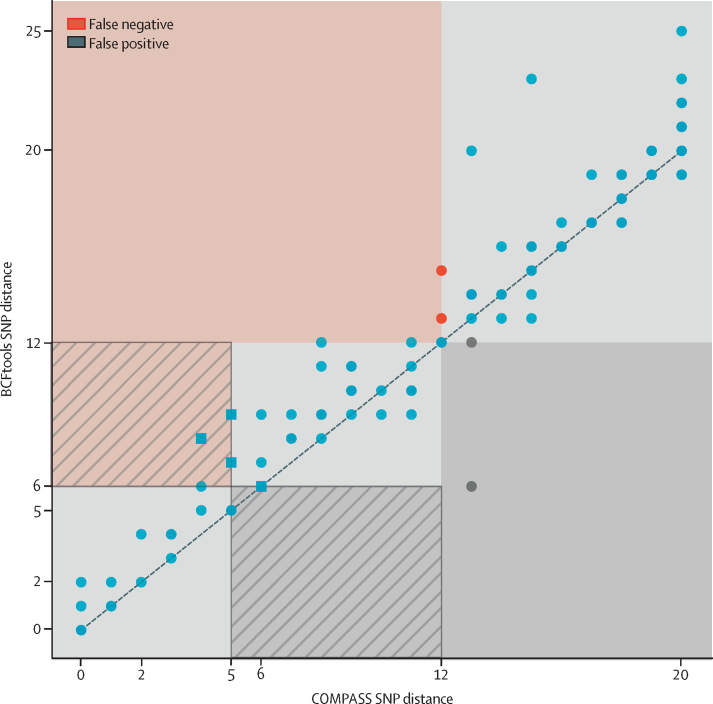
Pairwise SNP distance relationship between Illumina (COMPASS) and Nanopore (BCFtools) data Each point represents the SNP distance between two isolates. The dashed line shows the identity line (ie, y=x). The isolate pairs shown are all pairs whereby the COMPASS distance is 20 or less. The red area and points indicate pairs with a Nanopore distance of more than 12 but an Illumina distance of 12 or less. These pairs are deemed false negative connections. The red area with stripes indicates pairs that are false negative connections at an Illumina threshold of five (Nanopore threshold of six), but not when the threshold is expanded to 12. These pairs are shown as square points. The grey area and points are the inverse—ie, false positive connections. Thus, the grey striped area shows pairs of samples that are false positive connections at an Illumina threshold of five (Nanopore threshold six), but not when the threshold is expanded to 12. SNP=single-nucleotide polymorphism.

After applying these filters to all 151 study isolates, the SNP distances between all pairs of isolates as measured by COMPASS on the Illumina data, and our BCFtools pipeline on the Nanopore data, are significantly correlated (*R*^2^=0·988, p<0·0001; appendix p 18). The distance correlation for those isolates within 20 Illumina SNPs of each other (ie, the isolates most relevant to transmission investigations) is shown in figure 2. Encouragingly, at a distance threshold of 12, only two pairs of isolates (red points) were not linked by Nanopore, although we later showed that this only causes one isolate to be missed from its wider clustering.

We compared the clusters obtained from Illumina COMPASS and Nanopore BCFtools SNP calls using single-linkage clustering with the standard five-SNP and 12-SNP thresholds previously reported to correspond to highly and moderately probable transmission events.^16^, ^18^, ^19^ We developed three metrics (SACR, SACP, and XCR) to provide a quantitative assessment of how Nanopore clusters differed from baseline Illumina ones. Illumina and Nanopore SNP distances do not lie exactly on *y*=*x* (figure 2), and so we needed to use slightly different Nanopore SNP thresholds to match Illumina results. For Illumina thresholds of five and 12, we selected the respective Nanopore SNP threshold as follows. Because we sought to minimise the number of isolates missed from their true cluster, we chose the threshold which maximised SACR, under the constraint of not dropping SACP significantly (appendix p 21), there was a clear optimum in the curve, and we selected Nanopore SNP thresholds of six and 12.

**Figure 3:**
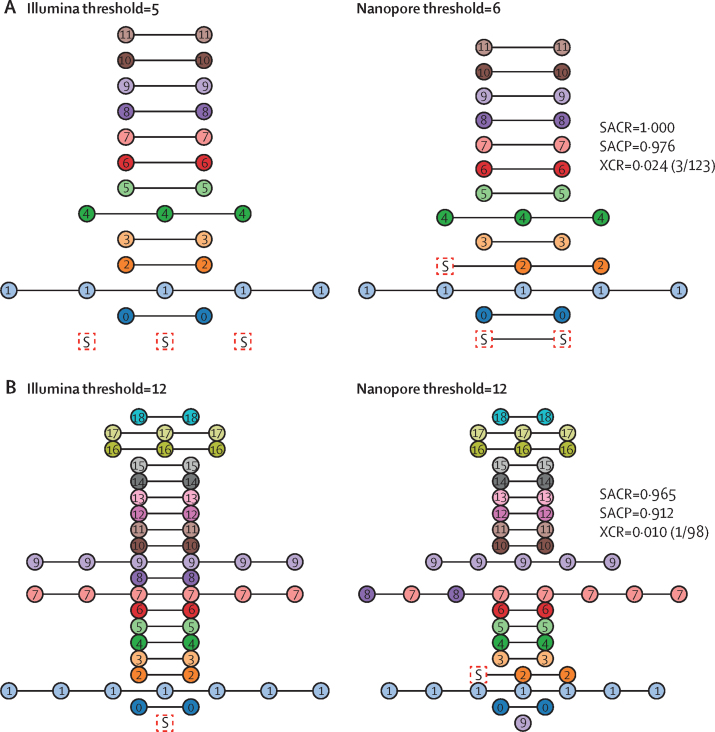
Agreement of Illumina and Nanopore transmission clustering with thresholds of five for Illumina and six for Nanopore (A) and thresholds of 12 for both technologies (B) The expected (Illumina COMPASS) clusters are shown on the left and the Nanopore BCFtools clustering is shown on the right. The title of each panel indicates the SNP threshold used for clustering. Nodes are coloured and numbered according to their Illumina cluster membership. Isolates clustered by Nanopore and not clustered (singletons) by Illumina are represented as boxes and are named S. Clusters are horizontally aligned and connected with black lines; however, the order of nodes and the length of edges have no significance. Each Nanopore panel shows the SACR, SACP, and XCR value (with the raw numbers in parentheses) with respect to the Illumina clustering. SACP=sample-averaged cluster precision. SACR=sample-averaged cluster recall. SNP=single-nucleotide polymorphism. XCR=excess clustering rate.

At these thresholds we found that isolates clustered together by Illumina remain clustered with Nanopore (nodes represent isolates and nodes of the same colour are connected), except at threshold 12, whereby Nanopore missed one isolate from cluster 9 (figure 3). At a threshold of five, the Illumina clusters are recapitulated, but one Illumina-singleton isolate is adjoined to cluster 2, and two Illumina-singleton isolates are combined into a new cluster. At threshold 12, clusters 7 and 8 are merged because Nanopore deemed two isolates to have a distance of 12 whereby the Illumina distance was 14. One isolate from cluster 9 is regarded a singleton by Nanopore; the severed connection had an Illumina distance of 12 and a Nanopore distance of 15. There was only one Illumina singleton adjoined to a pre-existing cluster (cluster 2) by Nanopore. For the thresholds of five for Illumina and six for Nanopore, the SACR value is 1·000, meaning Nanopore does not miss any isolates from their correct cluster. At a threshold of 12 for both technologies, the SACR is 0·965 and only one isolate was missed from its correct cluster. All clusters exclusively regrouped isolates from a same single country. Additionally, isolates from the same patient (n=8) were also clustered together by both technologies.

Next, we investigated isolate clustering when mixing sequencing modality data. As an initial check, the self-distance was calculated—ie, the SNP distance between the Nanopore-derived and Illumina-derived consensus genomic sequence for each isolate. The histogram of these values is shown in the appendix (p 23), confirming these distances were close to zero (mean 0·75 [SD 1·33]; median 0 [IQR 0·0–2·0]; appendix p 22). We also found the pairwise SNP distance of the mixed data to be significantly correlated with the Illumina distances (*R*^2^=0·992, p<0·0001; appendix p 24).

**Figure 4:**
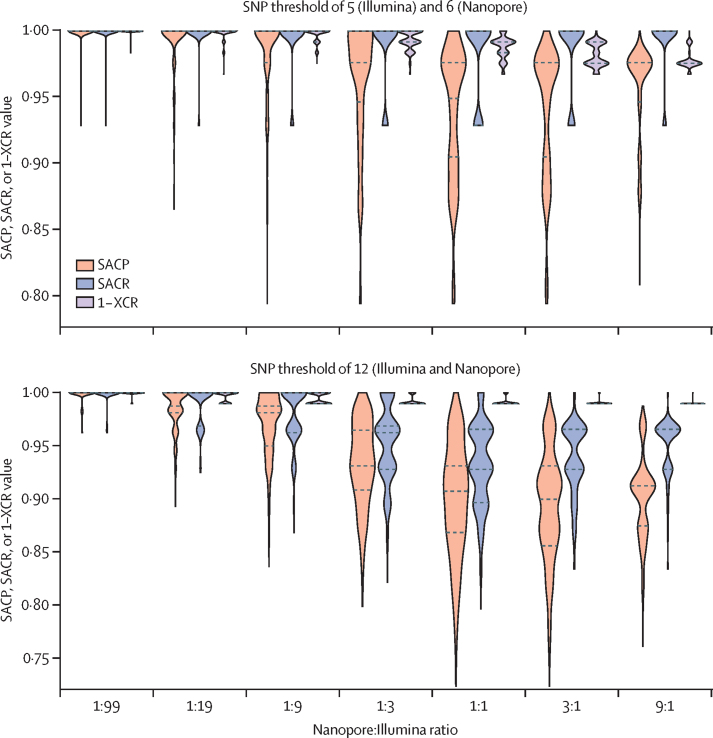
Simulating heterogeneous datasets with varying proportions of Nanopore and Illumina genomic data The different thresholds indicate the cutoff for defining isolates as part of a cluster. The y-axis depicts the SACP, SACR, or 1–XCR distributions over all simulation runs. For each ratio and threshold combination we ran 1000 simulations whereby the Nanopore and Illumina data were randomly split into the relevant ratio (eg, 1:9 means one Nanopore isolate for every nine Illumina isolates) and clusters were defined based on the relevant threshold. The titles for each subplot indicate the SNP threshold used when comparing Illumina, Nanopore, or mixed-technology isolate pairs. Dashed horizontal lines show the median and quartiles. SACP=sample-averaged cluster precision. SACR=sample-averaged cluster recall. SNP=single-nucleotide polymorphism. XCR=excess clustering rate.

Because our dataset consisted of 151 isolates with both Illumina and Nanopore data, we were able to simulate a wide range of mixed technology datasets by randomly assigning either the Nanopore or Illumina data to each isolate. We generated 1000 simulated datasets for each value in a range of Nanopore and Illumina ratios and measured the impact on SACR, SACP, and XCR (figure 4). As the proportion of Nanopore data increased, the recall (ie, SACR) was consistent, with the median fixed at 1·0 for a threshold of five. At the 12 SNP distance threshold the SACR lowers from 1·0 to a minimum (median) value of 0·928 (IQR 0·928–0·965) at a 3:1 Nanopore:Illumina ratio. The precision (SACP) degraded smoothly from 1·0 (meaning near-pure Illumina data perfectly recapitulate pure Illumina clusters) to a value similar to the pure Nanopore dataset (median of 0·949 [IQR 0·905–0·976] for the threshold of five and 0·899 [0·855–0·931] for the threshold of 12). The XCR gradually decreased to the pure Nanopore level.

To give some intuition on these metrics, we considered a simulated sample of 100 isolates including three clusters of size two, two, and nine, with 87 singletons. 50 were sequenced on Nanopore and the other 50 on Illumina. From these simulations, the expected SACR was 1·000, SACP was 0·847, and XCR was 0·031 for an SNP threshold of 12. The recall (ie, SACR) suggested that we would expect all isolates in our hypothetical dataset to be clustered with their expected cluster. The precision (ie, SACP) of 0·847 in this example would be equivalent to the two two-member clusters being joined into a single cluster, and an XCR value of 0·031 could be caused by three singleton isolates forming a new cluster.

We compared the Nanopore and Illumina drug resistance prediction genotype calls of Mykrobe at the 66 537 nucleotide-level resistance-conferring mutations for our 151 isolates and found four genotype discordances. Three of these discrepant mutations were *katG* 1 bp deletions at consecutive positions within a homopolymer in *katG,* all in the same isolate, effectively describing one deletion event and thus only affecting a single phenotype call. The other discrepancy was a *katG* 1 bp deletion in a separate isolate. There were also two further mutations (each in one isolate) that we did not classify as discrepant, in which a resistance mutation was detected with both Nanopore and Illumina, but filtered in the Illumina calls due to low coverage (*rrs* 1401A→G and *rrs* 514A→C; appendix pp 17–18). A summary of concordance of predictions is shown in the table. These results lead to a Cohen's κ statistic of 0·9915 (p<0·0001; appendix p 18), indicating near-perfect agreement, and the key observation for evaluating the utility of Nanopore data as a replacement or complement for Illumina for obtaining genotypic DST: if genotyping at resistance mutations is highly concordant (here 100% for SNPs and >99·99% for indels), then this concordance should be retained as catalogues of resistance mutations are improved.

**Table:** Comparison of Nanopore-based and Illumina-based drug resistance predictions using Mykrobe

	**Number of false negatives (number of resistant isolates)**	**Number of false positives (number of Illumina susceptible isolates)**	**False negative rate**	**False positive rate**	**Positive predictive value**	**Negative predictive value**
Isoniazid	0 (81)	1 (70)	0·0% (0·0–4·5)	1·4% (0·3–7·7)	98·8% (93·4–99·8)	100·0% (94·7–100·0)
Rifampicin	0 (79)	0 (72)	0·0% (0·0–4·6)	0·0% (0·0–5·1)	100·0% (95·4–100·0)	100·0% (94·9–100·0)
Ethambutol	0 (54)	0 (97)	0·0% (0·0–6·6)	0·0% (0·0–3·8)	100·0% (93·4–100·0)	100·0% (96·2–100·0)
Pyrazinamide	0 (30)	0 (121)	0·0% (0·0–11·4)	0·0% (0·0–3·1)	100·0% (88·6–100·0)	100·0% (96·9–100·0)
Streptomycin	0 (47)	1 (104)	0·0% (0·0–7·6)	1·0% (0·2–5·2)	97·9% (89·1–99·6)	100·0% (96·4–100·0)
Amikacin	0 (13)	1 (138)	0·0% (0·0–22·8)	0·7% (0·1–4·0)	92·9% (68·5–98·7)	100·0% (97·3–100·0)
Capreomycin	0 (13)	1 (138)	0·0% (0·0–22·8)	0·7% (0·1–4·0)	92·9% (68·5–98·7)	100·0% (97·3–100·0)
Kanamycin	0 (14)	1 (137)	0·0% (0·0–21·5)	0·7% (0·1–4·0)	93·3% (70·2–98·8)	100·0% (97·3–100·0)
Ciprofloxacin	0 (16)	0 (135)	0·0% (0·0–19·4)	0·0% (0·0–2·8)	100·0% (80·6–100·0)	100·0% (97·2–100·0)
Moxifloxacin	0 (16)	0 (135)	0·0% (0·0–19·4)	0·0% (0·0–2·8)	100·0% (80·6–100·0)	100·0% (97·2–100·0)
Ofloxacin	0 (17)	0 (134)	0·0% (0·0–18·4)	0·0% (0·0–2·8)	100·0% (81·6–100·0)	100·0% (97·2–100·0)

Data are % (95% CI) unless otherwise stated. For this comparison, we considered the Mykrobe resistance prediction from Illumina as the reference standard. A false negative means that Nanopore did not detect resistance but Illumina did. False positive means that Nanopore detected resistance but Illumina found susceptibility.

**Figure 5:**
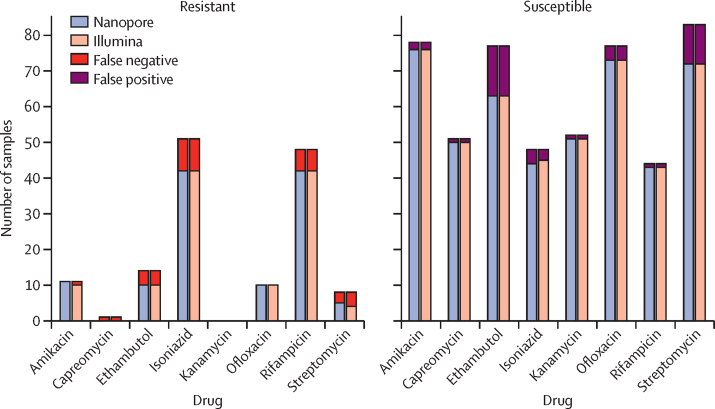
Number of resistant and susceptible phenotypes correctly predicted by Mykrobe from Illumina and Nanopore whole-genome sequencing data For resistant phenotypes (left plot) the bars show for each drug the breakdown of resistance predictions in samples that are phenotypically resistant; false negatives (wrongly calling a sample as susceptible) are coloured red, and the rest of the bar (true positives) is coloured to show the technology (Nanopore or Illumina). For susceptible phenotypes (right plot) the bars show for each drug the breakdown of resistance predictions in samples that are phenotypically susceptible; false positives (wrongly calling a sample as resistant) are coloured purple, and the rest of the bar (true negatives) is coloured to show the technology (Nanopore or Illumina).

For completeness, we showed the agreement of WGS predictions with available culture-based DST phenotypes (figure 5; appendix p 29). As expected, we saw that the Nanopore and Illumina results were nearly identical. Nanopore produced two fewer missed resistance (false negative) calls than Illumina (amikacin and streptomycin). However, Nanopore data lead to one extra false resistance (false positive) call compared with Illumina (isoniazid). Additionally, we saw no apparent improvement in prediction accuracy with increasing Nanopore read depth (appendix p 26).

## Discussion

The need for precision diagnostics supporting tuberculosis DST and transmission interruption is imperative. There is an increasing range of settings in which *M tuberculosis* genomic sequencing is deployed and progressively integrated within tuberculosis programmes and public health routine services. Although cases of drug resistance and transmission could motivate the adoption of tuberculosis WGS, the operational characteristics, costs, and analytical performance needs should be considered when committing to a sequencing platform. In this Article, we compare the performance of established Illumina and emerging Nanopore technologies in their ability to predict drug resistance and identify putative transmission clusters using SNPs (conditional on achieving at least 30× sequencing depth).

Effectiveness of a sequencing-based approach to these problems depends intrinsically on two factors. First, how well the genetic determinants of resistance are understood with respect to the various antitubercular drugs. Second, how well the sequence data from an isolate can be analysed to either detect all SNPs (used for clustering) or evaluate a list of known polymorphic positions (used for genotypic DST). The first question is technology independent and has been the subject of many studies over the past decade. Multiple studies have compiled catalogues of resistance mutations,^4^, ^10^, ^11^, ^20^ and in 2021 WHO published a knowledge base of high-confidence mutations intended to provide a solid foundation for future catalogues.^7^ Given perfect sequencing, the catalogue determines how well DST can be predicted, which is inexorably improving as the global community collects progressively more data.^21^, ^22^, ^23^, ^24^ In this study we take this trend as given, and ask whether Nanopore sequence data can provide as accurate genotyping of the resistance catalogue as Illumina data. If so, as catalogues improve, both Illumina and Nanopore technologies could be equally considered by reference laboratories developing their infrastructure and could be expected to provide concordant and progressively better results.

Our analysis shows that it is now possible to obtain high-precision SNP calls in *M tuberculosis* with current Nanopore data, with only a small decrease in recall—we obtained median precision of 99·3% and recall of 90·2% with Nanopore data, compared with a median precision of 99·6% and recall of 91·9% for Illumina. These results translate into six-SNP and 12-SNP Nanopore clusters, which are congruent with five-SNP and 12-SNP Illumina clusters. In terms of genotyping resistance-causing SNPs and indels, the two technologies give almost identical results using Mykrobe, with four discordances among 151 isolates multiplied by 66 537 nucleotide-level resistance-conferring mutations in the catalogue giving a concordance or more than 99·99%.

This study has limitations, particularly with regard to scope. First, we did not include epidemiological data to compare with the molecular clusters by design. Nevertheless, the genomic work we present here lays a foundation for investigating how Nanopore data perform with more nuanced approaches.^25^ Second, we restricted our analysis to whether Nanopore-based results from samples with good sequence depth (defined here as >30×) can match Illumina. It would be interesting to measure how performance degrades as depth drops well below 30× and to determine how far the multiplexing level could be increased while retaining acceptable results. Finally, it is worth emphasising that we were not able to draw conclusions on what proportion of samples can be successfully sequenced with Nanopore technology from this study; this conclusion would require a study design controlling for the level of multiplexing.

There has been a continual evolution and improvement of Nanopore data quality over the past 5 years. Our results evolved throughout the study as basecalling software and BCFtools were updated (data not shown). These updates required careful recalibration of variant filters. We strongly encourage validation of new software versions and flow cells, particularly basecalling and variant calling, and note that the data we present provide a valuable test set for this quality control.

The current momentum towards adoption of next-generation sequencing technologies for *M tuberculosis* genotypic DST and outbreak investigations is already well supported by WHO technical guidance and curated global mutation databases.^4^, ^6^, ^7^, ^18^, ^25^ Our work provides evidence to support the adoption of Nanopore sequencing, along with open access data and software which we hope will be of wide use. In our personal experience in Africa, the flexibility to analyse a single isolate (eg, when there is a suspected, extensively drug-resistant case) without batching is a major attraction of the technology.

**This online publication has been corrected. The corrected version first appeared at thelancet.com on December 22, 2022**

## Data sharing

The code used to perform all analyses and visualisations in this study is available at https://github.com/mbhall88/head_to_head_pipeline. We also make an open source (MIT licence) software workflow available at https://github.com/mbhall88/tbpore/ allowing others to analyse their own data. All sequencing reads generated for this study have been deposited in the European Nucleotide Archive under the project accession PRJEB49093. The raw Nanopore data are additionally available from https://ftp.ebi.ac.uk/pub/databases/ont_tb_eval2022/. A table listing accessions for each sample and run can be found at https://doi.org/10.6084/m9.figshare.19304648.

## Declaration of interests

ZI, SGL, and NR had travel and accommodation costs reimbursed when speaking at an Oxford Nanopore Technology (ONT) conference in 2017. SGL and NR previously received consumables from ONT when establishing Nanopore sequencing capacity in Madagascar. ONT matched the contributions from the Longitude Prize Discovery Award to ZI and TMW in 2017 to provide consumables for sequencing in Viet Nam and India. All other authors declare no competing interests. ONT did not provide funding (direct or in kind) for this project, and had no input or knowledge of the design, data analysis, or paper writing. Funders had no input into the design, data analysis, or paper writing of this project.
