# Correction to *Lancet Microbe* 2022; published online Dec 19, 2022. https://doi.org/10.1016/S2666-5247(22)00301-9

**DOI:** 10.1016/S2666-5247(22)00381-0

**Published:** 2023-02

*Hall MB, Rabodoarivelo MS, Koch A, et al. Evaluation of Nanopore sequencing for Mycobacterium tuberculosis drug susceptibility testing and outbreak investigation: a genomic analysis.* Lancet Microbe *2022; published online December 19. https://doi.org/10.1016/S2666-5247(22)00301-9*—In this Article, the Nanopore threshold in panel A of figure 3 should have read “6”. This correction has been made as of Dec 22, 2022.

