# Supplementary material for: Evaluation of Nanopore sequencing for Mycobacterium tuberculosis drug susceptibility testing and outbreak investigation: a genomic analysis
Source: Lancet Microbe. 2023 Feb;4(2):e84–92. doi: 10.1016/S2666-5247(22)00301-9 (PMC9892011; doi:10.1016/S2666-5247(22)00301-9)
Supplement: Supplementary appendix [file mmc1.pdf]

THE LANCET
Microbe

## Supplementary appendix

This appendix formed part of the original submission and has been peer reviewed. We post it as supplied by the authors.

Supplement to: Hall MB, Rabodoarivelo MS, Koch A, et al. Evaluation of Nanopore sequencing for *Mycobacterium tuberculosis* drug susceptibility testing and outbreak investigation: a genomic analysis. *Lancet Microbe* 2022; published online Dec 19. https://doi.org/10.1016/S2666-5247(22)00301-9.

# Supplementary information

## Contents

S1 Isolate selection and DNA extraction 5

S2 Extended sequencing methods 6

S3 Sequencing data preparation and quality control 8

S4 Variant calling 9

S5 Consensus genomic sequence assembly 10

S6 Extended clustering metrics definitions 11

S7 Illustrated example of clustering metrics 13

S8 Drug susceptibility testing methods 15

S9 Drug resistance predictions with Mykrobe 17

S10 Full dataset distance plot 18

S11 Selecting Nanopore SNP distance thresholds 20

S12 Mixed modality self-distance 22

S13 Mixed sequencing modality distance plot 24

S14 Effect of Nanopore read depth on resistance predictions 26

S15 Genotypic drug resistance prediction concordance with phenotype 28

References 30

# List of Figures

S1 Flowchart of the number of isolates we began (left) and ended (right) the quality control process with. Those lost to quality control are shown in red boxes. . . . . . . . . . . . . . . . . . . . 9
S2 Illustrative examples of transmission clustering. **a)** represents truth clusters, while **b)** is clustering from some "test" method we would like to compare to **a**. The nodes represent samples with the numbers on the edges connecting them indicating the distance between those two samples. The red nodes indicate samples with a clustering disparity between the two clusterings. Note, we do not show singletons (disconnected nodes) - e.g., $J$ is missing from **(b)**. . . . . . . . . . . . . . . . . . . . . . . . . . . . . . . . . 14
S3 Culture-based drug susceptibility data available for isolates. Each row is a drug, and the columns represent a set of isolates that have phenotype information for those drugs with a filled cell. The top panel shows the number of isolates in the set for that combination of drugs. The bar plot in the left panel shows the number of isolates with phenotype information for each drug. Phenotypic drug susceptibility testing was performed by clinical laboratories according to local testing algorithms which included complementary molecular testing and reflex sequential testing of second-line drugs. This explains why not all antibiotics were tested on all isolates. . . . . . . . . . . . . . . . . . . . . . . . . . 16
S4 Pairwise SNP distance relationship between Illumina (COMPASS; x-axis) and Nanopore (`bcftools`; y-axis). Each point represents the SNP distance between two isolates. The black, dashed line shows the identity line (i.e. $y = x$), while the red, dashed line shows the line of best fit to the data. $R^2$ is the coefficient of determination. . . . . . . . . . . . . . . . . . . . . . . . . . . 19
S5 Illumina and Nanopore (`bcftools`) transmission cluster similarity for various SNP distance threshold. Each subplot compares the Nanopore clustering for the threshold on the x-axis to the Illumina clustering based on the distance (threshold) in the subplot title. SACR (red), SACP (blue), and 1−XCR are represented by the solid, dashed, and dotted lines, respectively. SACR=sample-averaged cluster recall; SACP=sample-averaged cluster precision; XCR=excess clustering rate. . . . . . . . . . . . . . . . . . . . . 21
S6 Mixed modality "self-distance". This plot shows the SNP distance (x-axis) between each isolate's COMPASS (Illumina) and `bcftools` (Nanopore) VCF calls. Note, the y-axis is log-scaled. 23

S7 Pairwise SNP distance relationship between Illumina (COMPASS; x-axis) and mixed COMPASS-`bcftools` calls (y-axis). Each point represents the SNP distance between two isolates. The black, dashed line shows the identity line (i.e., y=x), while the red, dashed line shows the line of best fit to the data. $R^2$ is the coefficient of determination. The zoomed inset shows all pairs where the COMPASS distance is $\leq 20$. The red area and points indicate pairs with a mixed distance $\geq 12$, but an Illumina distance $\leq 12$. These pairs are deemed false negative (FN) connections. The red area with stripes indicates pairs that are FN connections at an Illumina threshold of 5 (mixed threshold 6), but not when the threshold is expanded to 12. These pairs are shown as square points. The grey area and points is the inverse - i.e., false positive (FP) connections. Note, the y-axis in the inset window is log-scaled. . . . . . . . . . . . . . . . . . . . . . . . . . . . . . . 25
S8 Effect of Nanopore read depth on Mykrobe DST prediction. Each point indicates the proportion (left y-axis) of classifications of that type at the read depth on the x-axis. The blue bars indicate the number of samples (right y-axis) contained in each bin. **(a)** read depth, $d$, is binned such that 40 is all samples where $40 \leq d \leq 50$. **(b)** read depth bin 40 is all samples where $d \geq 40$. FP=false positive (red); TP=true positive (blue); FN=false negative (purple); TN=true negative (grey). . . . . . . . . . . . . . . 27

# List of Tables

S1 Cluster recall and precision results for each sample in Figure S2. 14
S2 Comparison of WGS-based drug resistance predictions with culture-based phenotypes. For this comparison, we use phenotypic DST as the reference standard and evaluate Mykrobe Illumina and Nanopore resistance predictions accordingly. FN=false negative, meaning Nanopore does not detect resistance where Illumina does; R=number of resistant isolates; FP=false positive, meaning Nanopore detects resistance where Illumina finds susceptible; S=number of (Illumina) susceptible isolates; FNR=false negative rate; FPR=false positive rate; PPV=positive predictive value; NPV=negative predictive value; CI=Wilson score confidence interval. . . . . . . . . . . . . . . . . . . . . . . . . . . . . . . . . . . 29

S3 Discordances between Mykrobe genotype calls, and Mykrobe predicted phenotype when comparing Illumina and Nanopore. In the genotype column the Illumina and Nanopore genotype calls are shown separated by a forward-slash Illumina/Nanopore. F = call was filtered out; 0 = reference allele; 1 = alternate allele. The "Genotype discordant" column shows whether this difference was considered a discordance in our calculation. The "Phenotypes (predicted)" column shows the Mykrobe predictions for Illumina/Nanopore: S = susceptible; R = resistant. The "Phenotype discordant" column shows whether the predicted phenotypes in the previous column are the same. *The isolate had other mutations in *katG* that cause isoniazid resistance. †We skip mutations where either technology's call is filtered (see Section S9.1). 29

# S1 Isolate selection and DNA extraction

### Madagascar

The Institut Pasteur de Madagascar hosts the Madagascar Programme National de Lutte Contre la Tuberculose (National Tuberculosis Program), which provides reference drug susceptibility testing (DST) for tuberculosis (TB) patients suspected of facing infection relapse or treatment failure. Culture-confirmed multi-drug resistant (MDR) isolates were included. All MDR isolates were matched with culture-confirmed drug-susceptible isolates referred during the same period and from the same region of the country. All available isolates were included for patients undergoing TB treatment and for which a follow-up culture was positive.

DNA from *M. tuberculosis* culture was extracted using the cetyltrimethylammonium bromide (CTAB) method previously described by Van Embden *et al.* with minor modification.[1] Briefly, samples were inactivated by heating at 80°C for 30 minutes. Next, 10mg/ml of lysozyme was added to the suspension and incubated for at least one hour at 37°C. After adding proteinase K 10mg/ml and sodium dodecyl sulphate (SDS) 10%, the suspension was incubated for 10 minutes at 65°C. A mixture of CTAB and 5M NaCl preheated to 65°C was then added. The suspension was mixed until a milky mixture was obtained and incubated for 10 minutes at 65°C. Next, chloroform isoamyl alcohol (24:1) mixture was added, followed by centrifugation for 5 minutes at 10,000rpm and 4°C. The upper phase was recovered, and DNA was precipitated by adding isopropanol to the solution. The mixture was frozen for at least one hour then centrifuged at 10,000rpm and 4°C, for 5 minutes. The supernatant was discarded, and the pellet was washed with 70% ethanol and centrifuged at 10,000rpm and 4°C, for 5 minutes. The DNA pellet was dried with speed-vac for 2 minutes and resuspended in 1X TE. DNA was quantified using the Qubit dsDNA HS Assay Kit (Thermo Fisher Scientific, USA).

### South Africa

Clinical *M. tuberculosis* isolates routinely collected in the Western Cape Province of South Africa, processed by the National Health Laboratory Service (NHLS) and diagnosed as rifampicin-resistant TB, are biobanked at the South African Medical Research Council Centre for Tuberculosis Research (SAMRC-CTR) housed at the Division of Molecular Biology and Human Genetics at Stellenbosch University, South Africa. A convenience data set of 82 clinical *M. tuberculosis* isolates for which Illumina WGS data was available were selected for Nanopore sequencing.

Clinical *M. tuberculosis* isolates were cultured on supplemented 7H10 solid media under BSL3 conditions. Phenol-chloroform DNA extraction was performed on heat-inactivated cultured isolates as previously described.[2]

#### England

Total DNA was extracted from heat-inactivated, saline-washed Mycobacteria growth indicator tube (MGIT) cultures using two rounds of mechanical cell disruption, followed by DNA purification with 1x volume Agencourt AMPure XP beads, as described previously.[3]

# S2 Extended sequencing methods

## S2.1 Overview

Illumina sequencing was performed as per the manufacturer's instruction on either the MiSeq, HiSeq 2500, or NextSeq500 platforms. Nanopore sequencing was performed using the Ligation Sequencing Kit 1D (SQK-LSK108 or SQK-LSK109) and the Native Barcoding Kit 1D (EXP-NBD103 or EXP-NBD104) according to the manufacturer's instructions on either the MinION or GridION platform with R9·4·1 flow cells. In addition, 35 Malagasy isolates, including drug-resistant strains, were sequenced on the PacBio CCS platform.

## S2.2 Illumina

#### Madagascar

Illumina sequencing was carried out on the HiSeq 2500 platform at the Wellcome Trust Centre for Human Genetics, Oxford, and paired-end libraries were prepared according to the manufacturer's instruction.

#### England

Illumina sequencing was performed on a MiSeq instrument at Public Health England (Birmingham) by Grace Smith, Esther Robinson and their team. Sample preparation and sequencing methodology were as described previously.[3]

#### South Africa

Paired-end genomic libraries were prepared using the Illumina Nextera XT library or NEBNext Ultra TM II FS DNA Library Preparation Kits (Illumina Inc, San Diego, CA, USA) according to the manufacturers' instructions. Pooled samples were sequenced on an Illumina HiSeq2500 or NextSeq500 instrument.

## S2.3 Nanopore

#### Madagascar

Nanopore library preparation was carried out using the Oxford Nanopore Technology (ONT) Ligation Sequencing Kit 1D (SQK-LSK108) and the Native Barcoding Kit 1D (EXP-NBD103) according to the ONT standard protocols. One microgram of DNA was used as input for each library. Multiplexed sequencing

was performed by pooling 6-8 barcoded DNA samples. Prepared libraries were loaded onto an R9·4 flow cell and sequenced on a MinION device with ONT MinKNOW software.

#### England

Nanopore sequencing libraries were prepared using the 1D genomic kit (SQK-LSK108) with native barcoding kit (EXP-NBD103), according to the manufacturer's protocol. End-repaired, barcoded DNA ($\sim$ 8kb peak fragment lengths) from 3 to 7 samples were pooled into a single sequencing library and sequenced on a flow cell version R9·4·1 on either MinION or GridION.

#### South Africa

Remnant stored DNA used for Illumina WGS from each isolate was retrieved from storage and used for Nanopore library preparation. Per isolate, one microgram of undigested DNA was prepared for Nanopore sequencing using the ligation sequencing kit (SQK-LSK109). In addition, the native barcoding expansion kit (EXP-NBD104) was used for multiplexing. The protocols for sequencing genomic DNA by ligation and native barcoding were carried out according to the manufacturer's instructions. Multiplexed sequencing libraries consisted of 6-12 barcoded DNA samples, and all libraries were sequenced using SpotON R9·4·1 flow cells on a MinION device.

### S2.4 PacBio

Thirty-five of the Malagasy samples were sequenced and processed at the Next Generation Genomics Core within Cold Spring Harbor Laboratory. Samples were quantified with a Qubit dsDNA HS Assay Kit and quality controlled through a Pulsed Field Gel Electrophoresis system. Samples were then sheared at 10kb using a Megaruptor device and size-selected to 8-10 kb with a Blue pippin instrument - followed by 0·45X ampure bead purification. The PacBio library protocol SMRTbell Express Template Prep Kit 2·0 was used for each sample. Briefly, the first step was the removal of single-stranded overhangs followed by DNA Damage Repair, End-Repair/A-tailing, Ligation of overhang barcoded adaptors and sample pooling. A total of 3 pools were produced: LID50532 (16 samples), LID50533 (10 samples), and LID50534 (9 samples). After pooling, 0·5X ampure bead clean up was performed. A Sequel I instrument was used to sequence the 3 library pools. Libraries were annealed for an hour and bounded for an hour using sequel binding kit 3·0. Bound SMRTbell complexes were then purified with ampure beads. The run was set up as 10kb length for 10 hours movie time. The Sequel 1M V2 SMRT cells were used for each library.

The circular consensus was called via the SMRTlink graphical user interface version 6·0·0·47841.

# S3 Sequencing data preparation and quality control

## S3.1 Overview

Nanopore data were basecalled and de-multiplexed using the ONT software program Guppy (version 5·0·16). As a quality control, we removed non-*M. tuberculosis* sequencing reads by aligning all reads to a database of common contaminants.[4] Decontaminated data with mean read depth less than 20/30 (Illumina/Nanopore) were excluded from the study, as were isolates for which a single lineage could not be determined (as a proxy for potential contamination with a second strain). Figure S1 shows a summary of the number of isolates we began and ended the quality control process with.

## S3.2 Details

All Nanopore data were basecalled and de-multiplexed with ONT's software guppy (version 5·0·16) using the "super high-accuracy model" (`dna_r9.4.1_450bps_sup_prom.cfg`), disabling quality score filtering and with barcode trimming when de-multiplexing. Sequencing reads were first decontaminated using reference genomes from a wide range of organisms, including viral, human, Mycobacterial and nasopharyngeal-associated bacteria as described previously.[4] Then, any reads that aligned to the *M. tuberculosis* reference genome (accession: NC_000962.3; H37Rv) were retained. For Illumina, read-pairs with only one of the pair mapped to the reference were retained. All read mapping was performed using `minimap2` (v2·22)[5] for Nanopore and `bwa mem` (v0·7·17)[6] for Illumina. Isolates with decontaminated depth less than 30x (Nanopore) or 20x (Illumina) were removed from subsequent analyses. All remaining decontaminated fastq files were randomly subsampled to a depth of 150x (Nanopore) and 60x (Illumina) using `rasusa` (v0·6·0).[7] Any isolate with depth below this maximum threshold was left unchanged. In the last step of quality control (QC), lineages were assigned for each isolate. A panel of lineage-defining single nucleotide polymorphisms (SNPs) was used in conjunction with an isolate's Illumina variant calls for the lineage assignment as previously reported.[8] If a lineage could not be determined for an isolate, or if there were multiple (major) lineages identified (indicating mixture), the isolate was not included in the analysis.

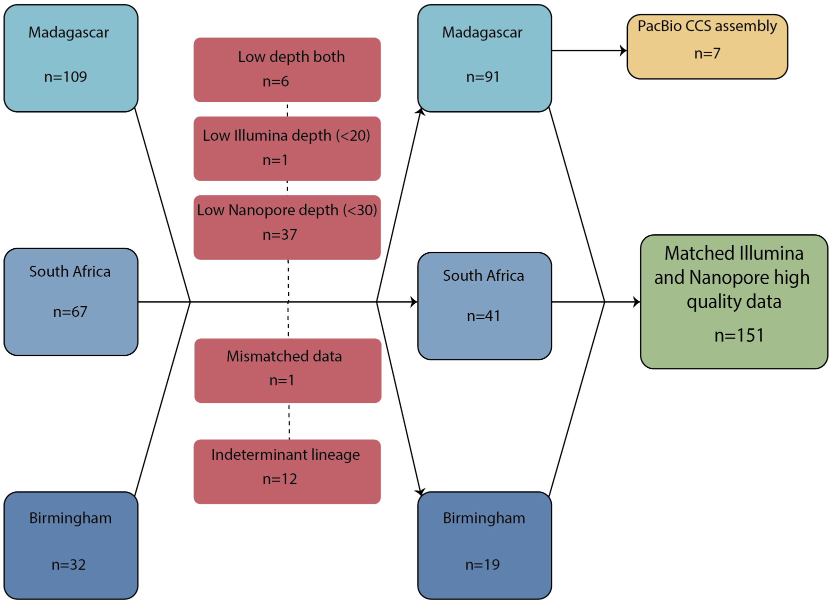


**Figure S1:** Flowchart of the number of isolates we began (left) and ended (right) the quality control process with. Those lost to quality control are shown in red boxes.

# S4 Variant calling

## S4.1 Illumina

Illumina variant calls were made using the COMPASS pipeline[9](https://github.com/oxfordmmm/CompassCompact) used by the United Kingdom Health Security Agency (UKHSA).[10] Briefly, reads were mapped to H37Rv (accession: NC_000962.3), and `samtools mpileup` (v0·1·19) was used to identify SNPs.[11] SNPs were filtered based on the following criteria: i) must have at least five high-quality ($> 25$) supporting bases, ii) must have at least one read in each direction, iii) 75% of reads must be high-quality, iv) the genotype, under a diploid model, must be homozygous, v) fraction of reads supporting the major allele must be at least 90%. In addition, any SNPs falling within a predefined masked region, defined by aligning H37Rv to itself and identifying repetitive regions[12], were excluded. The mask can be found at https://github.com/mbhall88/head_to_head_pipeline/blob/master/analysis/baseline_variants/resources/compass-mask.bed.

## S4.2 Nanopore

Nanopore reads were aligned to H37Rv using `minimap2` (v2·22), with options to produce SAM output and no secondary alignments (`-a --secondary=no`).

The subsequent SAM file was provided as input to the BCFtools (v1·13;[13]) subcommand `mpileup` with the following options: ignore read-pair overlaps (`-x`), do not perform insertion and deletion (indel) calling (`-I`), minimum base quality of 13 (`-Q 13`), a homopolymer error coefficient of 100 (`-h 100`), and a maximum read length of 10,000 for the BAQ algorithm (`-M 10000`). The resulting pileup was then used to call SNPs with `bcftools call` using the multi-allelic caller with a haploid model and an option to skip indel variants (`-m --ploidy 1 -V indels`).

Only SNPs passing the following criteria were kept: i) a quality score of at least 25, ii) each strand must have at least 1% of read depth, iii) read depth at least 20% of the isolate median read depth, iv) a variant distance bias (VDB; a measure of whether a variant's position is randomly distributed within the reads supporting it) of at least 0·00001, v) fraction of reads supporting the called allele of 90% or more, vi) read depth of at least 5 at the position, vii) mapping quality of at least 30. SNPs were also masked in the same manner as Illumina data outlined above (Section S4.1).

The selection of these filters was based on finding a balance between precision/recall in Section S5.1 - where we only have seven isolates - and the distance correlation with Illumina in Section S10.

The code for all of this can be found here: `https://github.com/mbhall88/head_to_head_pipeline/tree/master/analysis/baseline_variants`.

### S4.3 Pairwise SNP distances

To determine the distance between isolates, we first generated isolate consensus sequences for both Nanopore and Illumina sequencing modalities. This consensus sequence is obtained by applying the SNP calls (Section S4) to the *M. tuberculosis* reference genome H37Rv. We exclude any positions where i) the position failed filtering, ii) the genotype is null, or iii) the position is within the reference mask (Section S4.1). A pairwise distance matrix was calculated using `psdm` (v0·1·0).[14]

## S5 Consensus genomic sequence assembly

The eight PacBio isolates with read depth over 30x were assembled using Flye (v2·8)[15] with one polishing iteration and input type `--pacbio-hifi`. Assembly contigs were removed if they were classified by Centrifuge[16] as not being part of the *M. tuberculosis* complex (MTBC; taxon ID: 77643). During this contig decontamination process, one isolate was found to have chromosomes for three different species, and the PacBio data was therefore discarded leaving us with seven PacBio assemblies. We built a gold-standard reference sequence for each isolate using the unpolished PacBio assembly along with a mask for low-quality regions identified by aligning the isolates' Illumina reads to the PacBio assembly and flagging any position with either less than 10 reads mapping to it or less

than 90% agreement. These assemblies are used as a "truth" sequence for the respective isolates when assessing SNP precision and recall.

We selected PacBio CCS as the basis for these truth sequences as it has been shown to provide superior assemblies to Nanopore.[17] However, since there remain known issues for indel errors in PacBio assemblies (albeit at a lower rate than in nanopore) we remap Illumina data to the PacBio assemblies and mask out positions where there is disagreement, combining information from the highest quality assembly with the highest quality reads. The only possible remaining errors are in repeat sequence (where Illumina read mapping might fail) which are anyway excluded from our analysis by a fixed mask applied to the genome (Section S4.1). We thereby get as close as we can to the "truth".

### S5.1 Evaluation of precision and recall using PacBio assemblies as truth

Evaluation of precision and recall was done with `varifier` (v0·3·1)[18] to generate a list of true (expected) variants between the isolate's assembly and the *M. tuberculosis* reference sequence, and using the tool `hap.py` (v0·3·14)[19] to evaluate precision and recall.

## S6 Extended clustering metrics definitions

### S6.1 Outline

We treat Illumina as the established standard when comparing clustering. We define the Illumina clustering as $I$ and the Nanopore clustering as $N$. To quantify the recall and precision of the Nanopore clustering, we compared the clustering graphs $I$ and $N$ with three similarity metrics.

We define the sample-averaged cluster recall (SACR) by calculating, for each clustered isolate, $s$, in $I$, the proportion of isolates in its Illumina cluster ($C_{s,I}$) also present in its Nanopore cluster ($C_{s,N}$), and then averaging this over all isolates. We likewise define the sample-averaged cluster precision (SACP) as the proportion of isolates in $C_{s,N}$ also present in $C_{s,I}$ averaged over all isolates. SACR indicates whether isolates have been missed by Nanopore clustering (false negatives), and SACP reflects additional isolates being clustered by Nanopore (false positives).

One shortcoming of SACR and SACP is that they do not account for Nanopore clusters composed solely of Illumina singletons. Therefore, we define the excess clustering rate (XCR) as the proportion of Illumina singletons that are clustered by Nanopore. A value of 0·1 would indicate that 10 percent of Illumina singletons were part of a Nanopore cluster.

### S6.2 Details

As outlined above, to assess how closely Nanopore SNP-based clustering approximates Illumina SNP-based clustering, we adapt a similarity measure on sets;

the Tversky Index.[20] We define the Illumina clustering as $I$ and the Nanopore clustering as $N$. We are interested in being able to quantify the recall and precision of the Nanopore clustering with respect to Illumina. In this sense, recall describes the proportion of clustered samples (isolates) in $I$ clustered with the expected (correct) samples in $N$. Likewise, precision in this context tells us when extra samples are added to existing clusters by $N$ or when clusters in $I$ are joined in $N$.

In order to be able to define precision and recall when comparing two clustering graphs $I$ and $N$, we define the Tversky Index

$$TI(n, I, N) = \frac{|C_{n,I} \cap C_{n,N}|}{|C_{n,I} \cap C_{n,N}| + \alpha|C_{n,I} - C_{n,N}| + \beta|C_{n,N} - C_{n,I}|} \tag{S1}$$

where $C_{n,I}$ is the cluster in $I$ that sample $n$ is a member of. If $C_{n,I}$ is a singleton, $n$ is skipped. When $\alpha = 1$ and $\beta = 0$ in Equation S1, we get a metric analogous to recall - as described above. Therefore, we define recall, $R$, for a single sample $n$ as

$$R(n, I, N) = \frac{|C_{n,I} \cap C_{n,N}|}{|C_{n,I} \cap C_{n,N}| + |C_{n,I} - C_{n,N}|} = \frac{|C_{n,I} \cap C_{n,N}|}{|C_{n,I}|} \tag{S2}$$

When $\alpha = 0$ and $\beta = 1$ in Equation S1, we get a metric analogous to precision. As such, we define precision $P$, for a single sample $n$ as

$$P(n, I, N) = \frac{|C_{n,I} \cap C_{n,N}|}{|C_{n,I} \cap C_{n,N}| + |C_{n,N} - C_{n,I}|} = \frac{|C_{n,I} \cap C_{n,N}|}{|C_{n,N}|} \tag{S3}$$

With these definitions for a single sample, we can assess the recall and precision of the Nanopore clustering, $N$, with respect to the Illumina clustering, $I$, by averaging each metric over all samples in $I$. This gives us the *Sample-Averaged Cluster Recall* (SACR)

$$SACR = \frac{\sum_{n}^{V_I} R(n, I, N)}{|V_I|} \tag{S4}$$

where $V_I$ is the set of all non-singleton samples (nodes) in $I$ (Illumina graph). Likewise, we define the *Sample-Averaged Cluster Precision* (SACP) as

$$SACP = \frac{\sum_{n}^{V_I} P(n, I, N)}{|V_I|} \tag{S5}$$

SACR states, on average, what proportion of the samples clustered together in $I$ are also clustered together in $N$ (Nanopore) - it is a measure of how many true positives Nanopore retains. Inversely, SACP states, on average, what proportion of the samples clustered together in $N$ are also clustered together in $I$ - it is a measure of how many extra samples Nanopore adds to clusters.

However, SACR and SACP do not inherently account for when $N$ has clusters containing only samples deemed non-clustered (singleton) in $I$. In order

to quantify any extra clustering by $N$, we establish the *Excess Clustering Rate* (XCR) as the proportion of singletons (disconnected nodes) in $I$ that are connected in $N$. We define XCR as

$$XCR = \frac{|S_I - S_N|}{|S_I|} \tag{S6}$$

where $S_I$ and $S_N$ are the sets of singletons in the respective graphs.

In short, when trying to minimise the number of isolates missed from their cluster (as in the present work), it is desirable to improve SACR; for reducing over-clustering (false epidemiological links), focus on SACP; and when trying to reduce false clusters, one should aim to optimise XCR.
We assess the cluster similarities using the Python programming language with the `networkx` library.[21] For a given threshold, we create the Illumina clustering (graph), $I$, and the Nanopore clustering, $N$ - from the relevant distance matrix - and use these to calculate the SACR, SACP, and XCR using Equation S4, Equation S5, and Equation S6, respectively.

# S7 Illustrated example of clustering metrics

Section S6 outlines three metrics - SACR, SACP and XCR - for evaluating the similarity between two different strategies for transmission clustering. In order to provide the reader with greater intuition for the purpose of each metric, we present an illustrated example in Figure S2.

We take Figure S2a to be the truth clusters and Figure S2b to be test clusters. These are akin to Illumina ($I$) and Nanopore clusters ($N$), respectively, in Section S6. The individual recall and precision values (defined in Equation S2 and Equation S3) for each sample in Figure S2a are shown in Table S1. SACR and SACP (defined in Equation S4 and Equation S5) are *sample-averaged*, so their values for this example are 0·82 and 0·83 respectively.

To highlight the objective of SACR, we use the truth and test clusters containing the sample $F$. Samples $F$, $G$, $H$ and $I$ are shared between both, but $J$ is missing from the test cluster. To calculate the individual recall for $F$, we take the intersection size of the truth and test clusters it exists in and divide it by the size of the truth cluster - $\frac{4}{5} = 0{\cdot}8$. We do the same for the precision of sample $D$, except we divide by the size of the test cluster - giving $\frac{2}{3} = 0{\cdot}66$.

The relevance of the XCR metric is best exemplified by the test cluster containing samples $L$ and $M$. As we calculate SACR and SACP for all samples in the *truth* clusters, these two samples would be ignored. However, they are samples that - according to the truth - should not be part of any cluster (singletons). Therefore, SACR and SACP cannot capture these extra clusterings if they do not contain clustered truth samples. XCR covers this limitation and is the proportion of singletons in the truth that are clustered in the test (see Equation S6). As Figure S2 does not show singletons, let us pretend there are

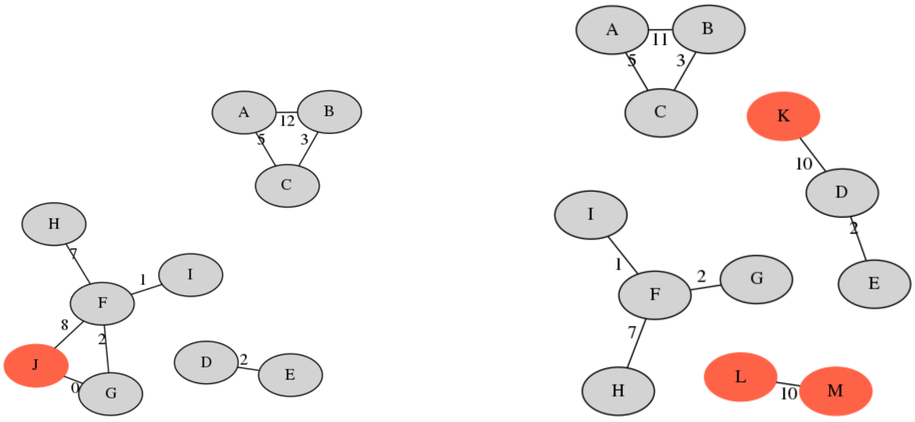


**(a)** Truth clusters

**(b)** Test clusters

**Figure S2:** Illustrative examples of transmission clustering. **a)** represents truth clusters, while **b)** is clustering from some "test" method we would like to compare to **a**. The nodes represent samples with the numbers on the edges connecting them indicating the distance between those two samples. The red nodes indicate samples with a clustering disparity between the two clusterings. Note, we do not show singletons (disconnected nodes) - e.g., $J$ is missing from **(b)**.

20 singletons in the truth (including samples $L$ and $M$). This would give an XCR of $\frac{2}{20} = 0{\cdot}1$.

| sample | recall | precision |
|---|---|---|
| A | 1·0 | 1·0 |
| B | 1·0 | 1·0 |
| C | 1·0 | 1·0 |
| D | 1·0 | 0·66 |
| E | 1·0 | 0·66 |
| F | 0·8 | 1·0 |
| G | 0·8 | 1·0 |
| H | 0·8 | 1·0 |
| I | 0·8 | 1·0 |
| J | 0·0 | 0·0 |
| sample-averaged | 0·82 | 0·83 |

**Table S1:** Cluster recall and precision results for each sample in Figure S2.

# S8 Drug susceptibility testing methods

## S8.1 Madagascar

Culture on Löwenstein-Jensen (LJ) is still the gold-standard method for *M. tuberculosis* identification and the detection of resistance. The indirect proportion method on LJ medium was performed to test the susceptibility of positive cultures against anti-*M. tuberculosis* drugs. $4\,\mu g\,mL^{-1}$, $0 \cdot 2\,\mu g\,mL^{-1}$, $40\,\mu g\,mL^{-1}$, $2\,\mu g\,mL^{-1}$, $30\,\mu g\,mL^{-1}$, $30\,\mu g\,mL^{-1}$, and $40\,\mu g\,mL^{-1}$ were the critical concentrations used for streptomycin, isoniazid, rifampicin, ethambutol, kanamycin, amikacin and capreomycin, respectively. The growth on a drug-free medium was compared with the growth on a medium containing an anti-*M. tuberculosis* agent. An isolate was identified as resistant if at least 1% of growth is present at the critical concentration of the drug in the culture medium.

## S8.2 South Africa

Isoniazid, ofloxacin, amikacin, and ethambutol (two concentrations) phenotypic DST was performed on Middlebrook 7H with critical concentrations $0 \cdot 2\,\mu g\,mL^{-1}$, $2 \cdot 0\,\mu g\,mL^{-1}$, $4 \cdot 0\,\mu g\,mL^{-1}$, and $7 \cdot 5\,\mu g\,mL^{-1}$ and $10 \cdot 0\,\mu g\,mL^{-1}$, respectively.

The phenotypes available for those isolates that passed quality control (Section S3) are shown in Figure S3. As an example of how to interpret Figure S3, the second "column" (from the left) reveals that 40 isolates have phenotype information for isoniazid, rifampicin, ethambutol, and streptomycin. Additionally, the second "row" (from the top) shows that 52 isolates have a DST phenotype for capreomycin.

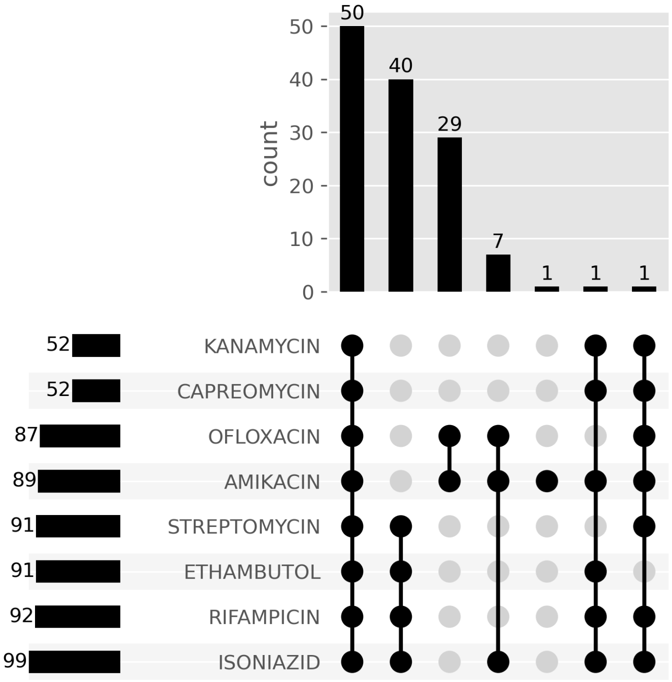


**Figure S3:** Culture-based drug susceptibility data available for isolates. Each row is a drug, and the columns represent a set of isolates that have phenotype information for those drugs with a filled cell. The top panel shows the number of isolates in the set for that combination of drugs. The bar plot in the left panel shows the number of isolates with phenotype information for each drug. Phenotypic drug susceptibility testing was performed by clinical laboratories according to local testing algorithms which included complementary molecular testing and reflex sequential testing of second-line drugs. This explains why not all antibiotics were tested on all isolates.

# S9 Drug resistance predictions with Mykrobe

We used Mykrobe (v0·10·0)[22] to obtain predictions of each isolates' drug susceptibility profile for 11 drugs - using the `predict` subcommand. For both technologies, we used a haploid model (`--ploidy haploid`) and a proportion of expected depth of 20% (`--min_proportion_expected_depth 0.2`). We set the expected error rate to 0·001 (`-e 0.001`) for Illumina and 0·08 (`-e 0.08`) for Nanopore. (We did not use the preset Nanopore settings (`--ont`).)

With Illumina data, Mykrobe can improve resistance predictions by detecting minor (low frequency, within-isolate) alleles, although this is not standard practice in the community. Including minor alleles resulted in elevated levels of discordance between Nanopore and Illumina, as many insertions and deletions were only (erroneously) detected with the Nanopore data. We therefore used a haploid model for both sequencing modalities.

## S9.1 Mutation concordance

We assessed the concordance of Mykrobe's Nanopore and Illumina genotypes for each of the 66,537 nucleotide-level mutations. In order to get genotypic information on all mutations in Mykrobe's catalogue, we ran Mykrobe with the `-A` option and `--format json` to output the results in the JSON format. For a given isolate, we compare the genotype of each mutation in both the Illumina and Nanopore JSON file. If there are any filters present (e.g., low coverage) for the mutation in either technology's output, we skip the mutation; otherwise, we consider the mutation concordant if the genotype is exactly the same between the two technologies.

In total, we found four genotype discordances (Table S3). Three of these discrepant mutations were *katG* 1bp deletions at consecutive positions within a homopolymer in *katG*, all in the same isolate, effectively describing one deletion event - thus only affecting a single phenotype call. The other discrepancy was a *katG* 1bp deletion in a separate isolate.

We then look at how these genotype differences translate into different predictions, which can differ subtly when there are no-calls (Table S3). There were five false positive (FP) resistance calls made by Mykrobe with Nanopore data, with respect to the Illumina predictions. Three of the FPs (amikacin, capreomycin, and kanamycin) are caused by one mutation (*rrs* a1401g) in a single isolate, which confers resistance to multiple drugs. There was also a discordant streptomycin call, due to *rrs* a514c. In both of these *rrs* FPs, the mutation was in fact detected by Illumina, but was filtered out due to low coverage (17 percent and 19 percent of expected coverage, respectively); the Nanopore data had very good depth for both of these mutations. This was not counted in the discordance count in the previous paragraph, as that counted variants where Illumina/Nanopore made actively conflicting genotype calls, whereas here there was no Illumina call (the call was filtered). There was also one isoniazid discordance, due to a frameshift deletion in *katG* (mentioned above) erroneously called by Nanopore. When consulting the DST phenotype for these discrep-

ancies, Nanopore had the correct predictions for the two *rrs* mutations, and Illumina had the correct prediction for the *katG* deletion. See Table S3 for a full description of the Mykrobe genotypic and phenotype prediction discrepancies.

### S9.2 Genotypic resistance prediction concordance

Table 1 in the main text shows the concordance of genotypic resistance predictions for Illumina and Nanopore.

To measure the agreement of the Illumina and Nanopore genotypic predictions, we calculated the Cohen's kappa coefficient ($\kappa$).[23] We aggregated predictions across all drugs for each sequencing technology into a 2x2 contingency matrix and supplied this to the `cohens_kappa` function in the python package `statsmodels`[24] to get $\kappa$ =0·9915 ($p$ <0·001), with a 95% confidence interval 0·9840-0·9989, indicating near perfect agreement (1·0 is the maximum value for $\kappa$).

## S10 Full dataset distance plot

Figure S4 shows the pairwise SNP distance relationship between Illumina and Nanopore. A two-sided linear least-squares regression was used to test the relationship between the pairwise SNP distance of both technologies with the Python package `scipy`.[25] The line of best fit (red, dashed line) was $y = 0{\cdot}997x + 15{\cdot}982$. The overall regression was statistically significant with a coefficient of determination ($R^2$) of 0·988 and $p < 0{\cdot}001$.

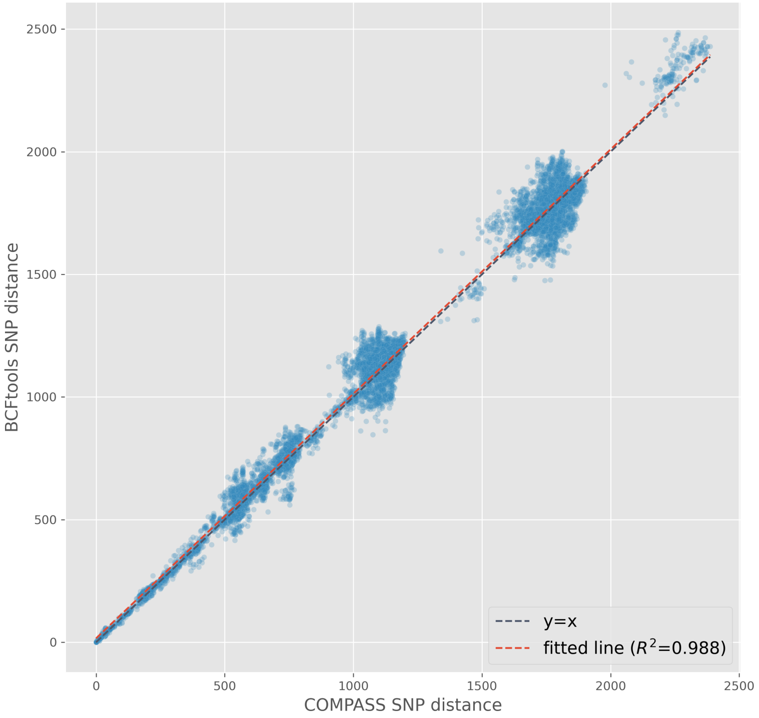


**Figure S4:** Pairwise SNP distance relationship between Illumina (COMPASS; x-axis) and Nanopore (`bcftools`; y-axis). Each point represents the SNP distance between two isolates. The black, dashed line shows the identity line (i.e. $y = x$), while the red, dashed line shows the line of best fit to the data. $R^2$ is the coefficient of determination.

## S11 Selecting Nanopore SNP distance thresholds

In order to select the Nanopore SNP distances that provide the most similar clustering to Illumina, we calculate the SACR, SACP, and XCR for a range of values (2-16). For the two Illumina distance thresholds 5 and 12, we create the Illumina clusters for the relevant distance threshold and compare those to the Nanopore clustering for each of values between 2 and 16. We then select the Nanopore threshold that provides the best balance of SACR, SAP, and XCR for the relevant Illumina threshold. Figure S5 shows the results of these threshold evaluations. Based on these, we select Nanopore distance thresholds 6 and 12 to correspond to Illumina thresholds 5 and 12, respectively.

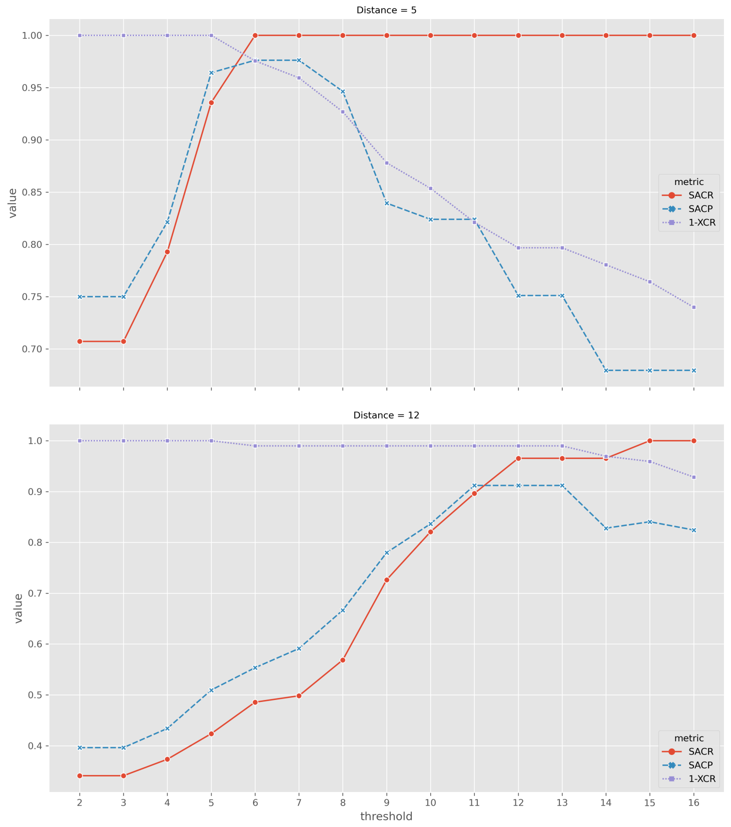


**Figure S5:** Illumina and Nanopore (`bcftools`) transmission cluster similarity for various SNP distance threshold. Each subplot compares the Nanopore clustering for the threshold on the x-axis to the Illumina clustering based on the distance (threshold) in the subplot title. SACR (red), SACP (blue), and 1−XCR are represented by the solid, dashed, and dotted lines, respectively. SACR=sample-averaged cluster recall; SACP=sample-averaged cluster precision; XCR=excess clustering rate.

# S12 Mixed modality self-distance

The "self-distance" for each isolate is the distance between an isolate's Illumina and Nanopore data. As the sequencing data in this study originate from the same source, we know the self-distance for any isolate *should* be 0. However, we also know there are major technical differences between Illumina and Nanopore; therefore, small variability in self-distance is likely. We plot the self-distances in Figure S6 and see that 68% (102/151) of the isolates have a distance of 0 between their Illumina (COMPASS) and Nanopore (`bcftools`) data, with 90% (136/151) less than 3 SNPs apart. All isolates have a self-distance of less than 5, except one isolate (`mada_1-33`), which has a self-distance of 10. We investigated the possibility of a sample mix-up being the cause of this discrepancy but could not find any such convincing evidence.

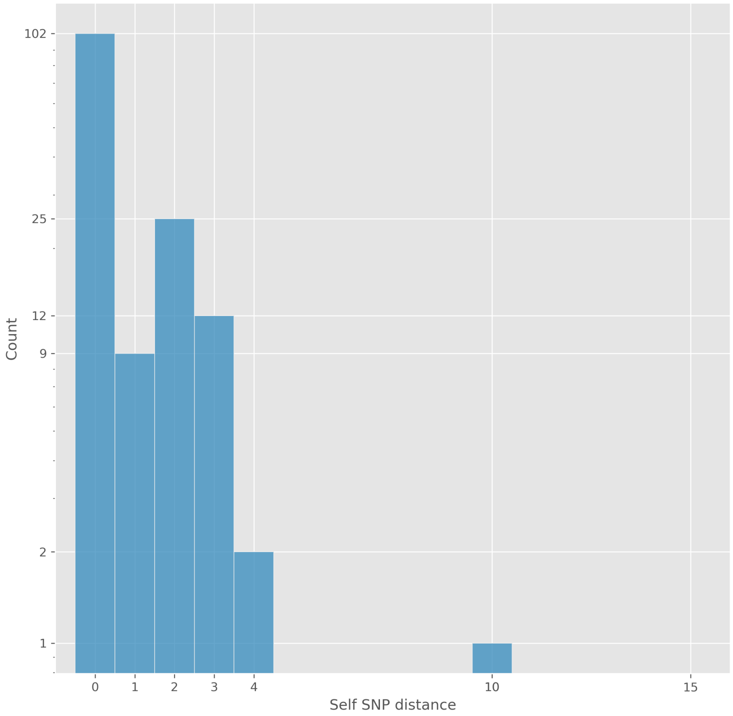


**Figure S6:** Mixed modality "self-distance". This plot shows the SNP distance (x-axis) between each isolate's COMPASS (Illumina) and `bcftools` (Nanopore) VCF calls. Note, the y-axis is log-scaled.

## S13 Mixed sequencing modality distance plot

Figure S7 shows the pairwise SNP distance relationship between Illumina and mixed Illumina/Nanopore data. A point on the y-axis (mixed) represents the distance between an isolate's Illumina consensus sequence, and the other isolate's Nanopore consensus sequence.

A two-sided linear least-squares regression was used to test the relationship between the pairwise SNP distance of mixed data with Illumina - using the Python package `scipy`.[25] The line of best fit (red, dashed line) was $y = 0{\cdot}995x + 14{\cdot}830$. The overall regression was statistically significant with a coefficient of determination ($R^2$) of 0·992 and $p < 0{\cdot}001$.

The outliers in the inset window of Figure S7 are due to discrepant SNP calls between some isolate's Illumina and Nanopore data in the gene *ppe54*. There are some gaps in the genome mask in this gene, and nearly all of the discrepancies lie within one of these gaps.

In general, what is happening is one isolate's Illumina calls good quality SNPs (or reference alleles) in these regions, but the same isolate's Nanopore has filtered calls for low quality and/or low fraction of read support for those sites. Then, the inverse happens in the other isolate - i.e., the Illumina generally has no coverage in the gaps but the Nanopore has good quality calls. So, when you do the same-technology distances (Figure 3 in the main text), these sites are all ignored due to filtering on one of the sequencing modalities, but when we assessing mixed-modality distance, these sites lead to larger-than-expected distances.

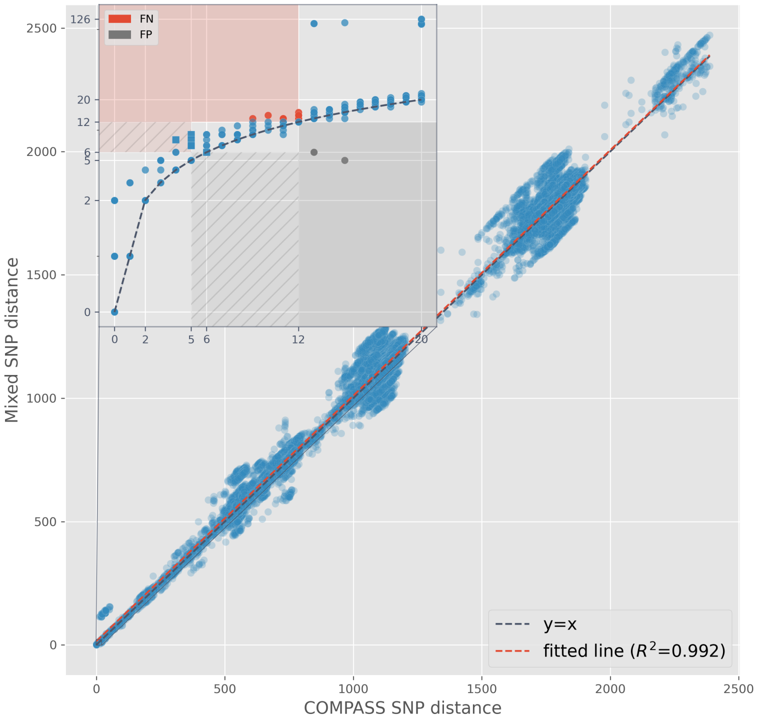


**Figure S7:** Pairwise SNP distance relationship between Illumina (COMPASS; x-axis) and mixed COMPASS-`bcftools` calls (y-axis). Each point represents the SNP distance between two isolates. The black, dashed line shows the identity line (i.e., y=x), while the red, dashed line shows the line of best fit to the data. $R^2$ is the coefficient of determination. The zoomed inset shows all pairs where the COMPASS distance is $\leq 20$. The red area and points indicate pairs with a mixed distance $\geq 12$, but an Illumina distance $\leq 12$. These pairs are deemed false negative (FN) connections. The red area with stripes indicates pairs that are FN connections at an Illumina threshold of 5 (mixed threshold 6), but not when the threshold is expanded to 12. These pairs are shown as square points. The grey area and points is the inverse - i.e., false positive (FP) connections. Note, the y-axis in the inset window is log-scaled.

# S14 Effect of Nanopore read depth on resistance predictions

An important consideration when using Nanopore sequencing data for DST prediction is how much data is needed. The quantity of data required has implications for how long the Nanopore sequencing device needs to be run or how many samples can be multiplexed in a single run in order to yield sufficient data for reliable predictions. A previous study by Votintseva *et al.* found that deep coverage is required of Nanopore to predict drug resistance accurately.[26]

As our dataset contains a broad range of Nanopore read depths (29-150x), we explore whether this requirement of high coverage still holds. As Figure S8 illustrates, there is no relationship between Nanopore read depth and erroneous predictions. If low read depth leads to poor resistance prediction, we would expect the proportions of FPs (red) and FNs (purple) in the low-depth bins to be greater than in the high-depth bins - which is not the case.

Given these results, we found no clear evidence that depth as low as 30x leads to an increased level of false phenotype predictions. To put this low-depth result into perspective, we can do a "back of the envelope" calculation to determine how long a Nanopore device needs to be operated to achieve 30x coverage theoretically. First, we use a conservative yield from Smith *et al.* of 11 gigabases (total) per 24 hours with six samples multiplexed on a single flow cell.[27] Assuming equal depth from each sample, this equates to 76·4Mb/hour/sample - dividing by the *M. tuberculosis* genome size (4·41Mb) gives an hourly coverage yield of 17·3x per sample. Finally, we divide our desired yield, 30x, by this per-sample hourly yield to get a required device runtime of 1 hour and 45 minutes. In contrast, to get 100x of data (the target coverage in [27]), the Nanopore device would need to be run for 5 hours and 47 minutes. Thus, in theory, one can save 4 hours of sequencing, use more samples per flow cell, decrease costs as a result, and still obtain reliable drug resistance predictions.

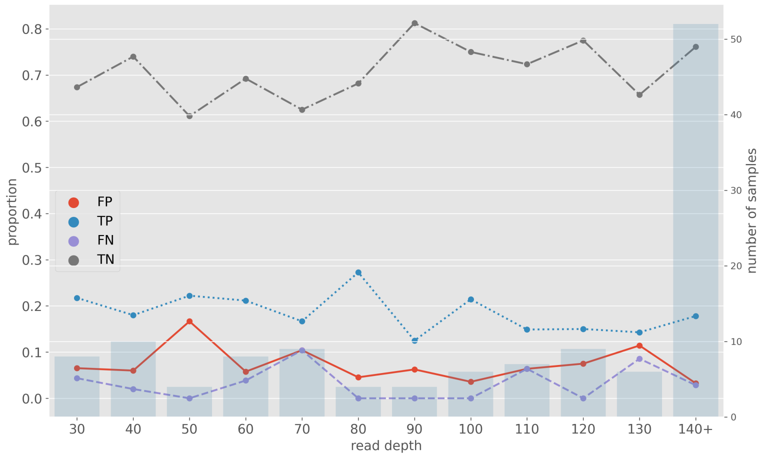


**(a)**

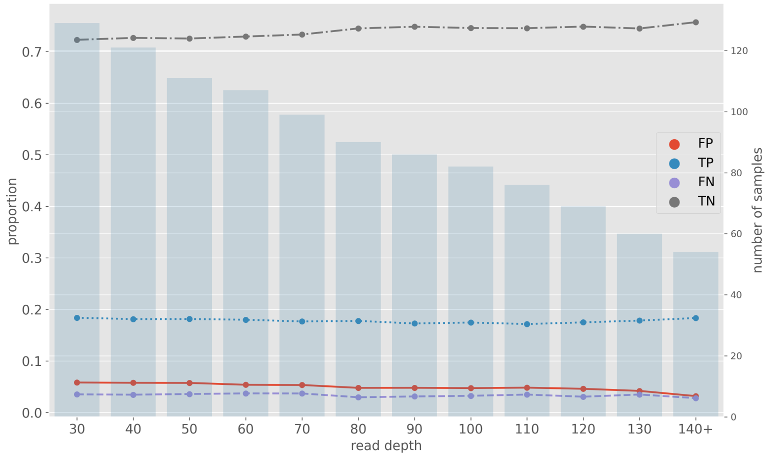


**(b)**

**Figure S8:** Effect of Nanopore read depth on Mykrobe DST prediction. Each point indicates the proportion (left y-axis) of classifications of that type at the read depth on the x-axis. The blue bars indicate the number of samples (right y-axis) contained in each bin. **(a)** read depth, $d$, is binned such that 40 is all samples where $40 \leq d \leq 50$. **(b)** read depth bin 40 is all samples where $d \geq 40$. FP=false positive (red); TP=true positive (blue); FN=false negative (purple); TN=true negative (grey).

## S15 Genotypic drug resistance prediction concordance with phenotype

| Drug | Technology | FN(R) | FP(S) | FNR(95% CI) | FPR(95% CI) | PPV(95% CI) | NPV(95% CI) |
|---|---|---|---|---|---|---|---|
| Isoniazid | Illumina | 9(51) | 3(48) | 17·6% (9·6-30·3%) | 6·2% (2·1-16·8%) | 93·3% (82·1-97·7%) | 83·3% (71·3-91·0%) |
| Isoniazid | Nanopore | 9(51) | 4(48) | 17·6% (9·6-30·3%) | 8·3% (3·3-19·6%) | 91·3% (79·7-96·6%) | 83·0% (70·8-90·8%) |
| Rifampicin | Illumina | 6(48) | 1(44) | 12·5% (5·9-24·7%) | 2·3% (0·4-11·8%) | 97·7% (87·9-99·6%) | 87·8% (75·8-94·3%) |
| Rifampicin | Nanopore | 6(48) | 1(44) | 12·5% (5·9-24·7%) | 2·3% (0·4-11·8%) | 97·7% (87·9-99·6%) | 87·8% (75·8-94·3%) |
| Ethambutol | Illumina | 4(14) | 14(77) | 28·6% (11·7-54·6%) | 18·2% (11·2-28·2%) | 41·7% (24·5-61·2%) | 94·0% (85·6-97·7%) |
| Ethambutol | Nanopore | 4(14) | 14(77) | 28·6% (11·7-54·6%) | 18·2% (11·2-28·2%) | 41·7% (24·5-61·2%) | 94·0% (85·6-97·7%) |
| Streptomycin | Illumina | 4(8) | 11(83) | 50·0% (21·5-78·5%) | 13·3% (7·6-22·2%) | 26·7% (10·9-52·0%) | 94·7% (87·2-97·9%) |
| Streptomycin | Nanopore | 3(8) | 11(83) | 37·5% (13·7-69·4%) | 13·3% (7·6-22·2%) | 31·2% (14·2-55·6%) | 96·0% (88·9-98·6%) |
| Amikacin | Illumina | 1(11) | 2(78) | 9·1% (1·6-37·7%) | 2·6% (0·7-8·9%) | 83·3% (55·2-95·3%) | 98·7% (93·0-99·8%) |
| Amikacin | Nanopore | 0(11) | 2(78) | 0·0% (0·0-25·9%) | 2·6% (0·7-8·9%) | 84·6% (57·8-95·7%) | 100·0% (95·2-100·0%) |
| Capreomycin | Illumina | 1(1) | 1(51) | 100·0% (20·7-100·0%) | 2·0% (0·3-10·3%) | 0·0% (0·0-79·3%) | 98·0% (89·7-99·7%) |
| Capreomycin | Nanopore | 1(1) | 1(51) | 100·0% (20·7-100·0%) | 2·0% (0·3-10·3%) | 0·0% (0·0-79·3%) | 98·0% (89·7-99·7%) |
| Kanamycin | Illumina | 0(0) | 1(52) | - | 1·9% (0·3-10·1%) | 0·0% (0·0-79·3%) | 100·0% (93·0-100·0%) |
| Kanamycin | Nanopore | 0(0) | 1(52) | - | 1·9% (0·3-10·1%) | 0·0% (0·0-79·3%) | 100·0% (93·0-100·0%) |
| Ofloxacin | Illumina | 0(10) | 4(77) | 0·0% (-0·0-27·8%) | 5·2% (2·0-12·6%) | 71·4% (45·4-88·3%) | 100·0% (95·0-100·0%) |
| Ofloxacin | Nanopore | 0(10) | 4(77) | 0·0% (-0·0-27·8%) | 5·2% (2·0-12·6%) | 71·4% (45·4-88·3%) | 100·0% (95·0-100·0%) |

**Table S2:** Comparison of WGS-based drug resistance predictions with culture-based phenotypes. For this comparison, we use phenotypic DST as the reference standard and evaluate Mykrobe Illumina and Nanopore resistance predictions accordingly. FN=false negative, meaning Nanopore does not detect resistance where Illumina does; R=number of resistant isolates; FP=false positive, meaning Nanopore detects resistance where Illumina finds susceptible; S=number of (Illumina) susceptible isolates; FNR=false negative rate; FPR=false positive rate; PPV=positive predictive value; NPV=negative predictive value; CI=Wilson score confidence interval.

| Isolate | Mutation | Genotype | Genotype discordant | Phenotype (predicted) | Phenotype discordant |
|---|---|---|---|---|---|
| `mada_135` | *katG* gc1037c | 0 / 1 | YES | S / R | YES |
| `mada_135` | *katG* cc1038c | 0 / 1 | YES | S / R | YES |
| `mada_135` | *katG* cc1039c | 0 / 1 | YES | S / R | YES |
| `mada_1-41` | *katG* cc1038c | 0 / 1 | YES | R* / R | NO* |
| `R26791` | *rrs* a1401g | F / 1 | NO† | S / R | YES |
| `mada_1-3` | *rrs* a514c | F / 1 | NO† | S / R | YES |

**Table S3:** Discordances between Mykrobe genotype calls, and Mykrobe predicted phenotype when comparing Illumina and Nanopore. In the genotype column the Illumina and Nanopore genotype calls are shown separated by a forward-slash Illumina/Nanopore. F = call was filtered out; 0 = reference allele; 1 = alternate allele. The "Genotype discordant" column shows whether this difference was considered a discordance in our calculation. The "Phenotypes (predicted)" column shows the Mykrobe predictions for Illumina/Nanopore: S = susceptible; R = resistant. The "Phenotype discordant" column shows whether the predicted phenotypes in the previous column are the same.

*The isolate had other mutations in *katG* that cause isoniazid resistance.

†We skip mutations where either technology's call is filtered (see Section S9.1).

# References


[1] J D van Embden et al. "Strain identification of Mycobacterium tuberculosis by DNA fingerprinting: recommendations for a standardized methodology". In: *Journal of Clinical Microbiology* 31.2 (1993), pp. 406–409. DOI: 10.1128/jcm.31.2.406-409.1993.

[2] Robin Warren et al. "Safe Mycobacterium tuberculosis DNA extraction method that does not compromise integrity". In: *Journal of clinical microbiology* 44.1 (Jan. 2006), pp. 254–256. ISSN: 0095-1137. DOI: 10.1128/jcm.44.1.254-256.2006.

[3] Antonina A. Votintseva et al. "Mycobacterial DNA Extraction for Whole-Genome Sequencing from Early Positive Liquid (MGIT) Cultures". In: *Journal of Clinical Microbiology* 53.4 (2015), pp. 1137–1143. ISSN: 0095-1137. DOI: 10.1128/jcm.03073-14.

[4] Alice Brankin et al. "A data compendium of Mycobacterium tuberculosis antibiotic resistance". In: *bioRxiv* (2021). DOI: 10.1101/2021.09.14.460274.

[5] Heng Li. "Minimap2: pairwise alignment for nucleotide sequences". In: *Bioinformatics* 34.18 (2018), pp. 3094–3100. ISSN: 1367-4803. DOI: 10.1093/bioinformatics/bty191.

[6] Heng Li. *Aligning sequence reads, clone sequences and assembly contigs with BWA-MEM*. 2013. arXiv: 1303.3997 [q-bio.GN].

[7] Michael B. Hall. "Rasusa: Randomly subsample sequencing reads to a specified coverage". In: *Journal of Open Source Software* 7.69 (2022), p. 3941. DOI: 10.21105/joss.03941.

[8] Marni LaFleur et al. "Drug-Resistant Tuberculosis in Pet Ring-Tailed Lemur, Madagascar". In: *Emerging Infectious Diseases* 27.3 (Mar. 2021), pp. 977–979. DOI: 10.3201/eid2703.202924.

[9] Rana Jajou et al. "Towards standardisation: comparison of five whole genome sequencing (WGS) analysis pipelines for detection of epidemiologically linked tuberculosis cases". In: *Eurosurveillance* 24.50, 1900130 (2019). DOI: 10.2807/1560-7917.ES.2019.24.50.1900130.

[10] Timothy M Walker et al. "Tuberculosis is changing". In: *The Lancet Infectious Diseases* 17.4 (Apr. 2017), pp. 359–361. DOI: 10.1016/s1473-3099(17)30123-8.

[11] Heng Li et al. "The Sequence Alignment/Map format and SAMtools". In: *Bioinformatics* 25.16 (2009), pp. 2078–2079. ISSN: 1367-4803. DOI: 10.1093/bioinformatics/btp352.

[12] Timothy M Walker et al. "Assessment of Mycobacterium tuberculosis transmission in Oxfordshire, UK, 2007–12, with whole pathogen genome sequences: an observational study". In: *The Lancet Respiratory Medicine* 2.4 (2014), pp. 285–292. ISSN: 2213-2600. DOI: 10.1016/s2213-2600(14)70027-x.

[13] Petr Danecek et al. "Twelve years of SAMtools and BCFtools". In: *GigaScience* 10.2 (2021), giab008. ISSN: 2047-217X. DOI: 10.1093/gigascience/giab008.

[14] Michael B. Hall. *psdm: Compute a pairwise SNP distance matrix from one or two alignment(s)*. Version 0.1.0. Nov. 2021. DOI: 10.5281/zenodo.5706785.

[15] Mikhail Kolmogorov et al. "Assembly of long, error-prone reads using repeat graphs". In: *Nature Biotechnology* 37.5 (Apr. 2019), pp. 540–546. DOI: 10.1038/s41587-019-0072-8.

[16] Daehwan Kim et al. "Centrifuge: rapid and sensitive classification of metagenomic sequences". In: *Genome Research* 26.12 (Oct. 2016), pp. 1721–1729. DOI: 10.1101/gr.210641.116.

[17] Aaron M. Wenger et al. "Accurate circular consensus long-read sequencing improves variant detection and assembly of a human genome". In: *Nature Biotechnology* 37.10 (2019), pp. 1155–1162. ISSN: 1087-0156. DOI: 10.1038/s41587-019-0217-9.

[18] Rachel M. Colquhoun et al. "Pandora: nucleotide-resolution bacterial pan-genomics with reference graphs". In: *Genome Biology* 22.1 (Sept. 2021), p. 267. ISSN: 1474-760X. DOI: 10.1186/s13059-021-02473-1.

[19] Peter Krusche et al. "Best practices for benchmarking germline small-variant calls in human genomes". In: *Nature Biotechnology* 37.5 (2019), pp. 555–560. ISSN: 1087-0156. DOI: 10.1038/s41587-019-0054-x.

[20] Amos Tversky. "Features of similarity". In: *Psychological Review* 84.4 (1977), pp. 327–352. ISSN: 0033-295X. DOI: 10.1037/0033-295x.84.4.327.

[21] Aric A. Hagberg, Daniel A. Schult, and Pieter J. Swart. "Exploring Network Structure, Dynamics, and Function using NetworkX". In: *Proceedings of the 7th Python in Science Conference*. Ed. by Gaël Varoquaux, Travis Vaught, and Jarrod Millman. Pasadena, CA USA, 2008, pp. 11–15. URL: https://www.osti.gov/biblio/960616.

[22] Martin Hunt et al. "Antibiotic resistance prediction for Mycobacterium tuberculosis from genome sequence data with Mykrobe". In: *Wellcome Open Research* 4 (2019), p. 191. ISSN: 2398-502X. DOI: 10.12688/wellcomeopenres.15603.1.

[23] Jacob Cohen. "A Coefficient of Agreement for Nominal Scales". In: *Educational and Psychological Measurement* 20.1 (1960), pp. 37–46. ISSN: 0013-1644. DOI: 10.1177/001316446002000104.

[24] Skipper Seabold and Josef Perktold. "statsmodels: Econometric and statistical modeling with python". In: *9th Python in Science Conference*. 2010.

[25] Pauli Virtanen et al. "SciPy 1.0: Fundamental Algorithms for Scientific Computing in Python". In: *Nature Methods* 17 (2020), pp. 261–272. DOI: 10.1038/s41592-019-0686-2.

[26] Antonina A. Votintseva et al. "Same-Day Diagnostic and Surveillance Data for Tuberculosis via Whole-Genome Sequencing of Direct Respiratory Samples". In: *Journal of Clinical Microbiology* 55.5 (2017), pp. 1285–1298. ISSN: 0095-1137. DOI: 10.1128/jcm.02483-16.

[27] Carol Smith et al. "Assessing Nanopore sequencing for clinical diagnostics: A comparison of NGS methods for Mycobacterium tuberculosis". In: *Journal of Clinical Microbiology* (2020). ISSN: 0095-1137. DOI: 10.1128/jcm.00583-20.
